# Mechanical Strain‐Programmed SDC1^+^ Sheath Fibroblasts Trigger CXCR4^hi^ Neutrophil‐Mediated Enthesitis in Ankylosing Spondylitis

**DOI:** 10.1002/advs.202520617

**Published:** 2026-02-04

**Authors:** Jiajie Lin, Zepeng Su, Yipeng Zeng, Yi Zhou, Chenying Zeng, Weihao Zhang, Qibo Li, Zipeng Xiao, Zibin Chen, Ziqian Liu, Yangfeng Lin, Guan Zheng, Wenhui Yu, Zhongyu Xie

**Affiliations:** ^1^ Department of Orthopedics The Eighth Affiliated Hospital Sun Yat‐sen University Shenzhen P. R. China; ^2^ Center for Biotherapy The Eighth Affiliated Hospital Sun Yat‐sen University Shenzhen P. R. China; ^3^ Guangdong Provincial Clinical Research Center for Orthopedic Diseases Shenzhen P. R. China

**Keywords:** ankylosing spondylitis, enthesitis, fibroblast, mechanical strain, neutrophil

## Abstract

Ankylosing spondylitis (AS) is an osteoimmune disease characterized by pathological enthesitis related to mechanical strain. However, the cell interactions and molecular mechanisms of AS enthesitis are still unclear. Herein, we constructed a hind paw loading/unloading model using experimental spondyloarthritis SKG mice, and generated a single‐cell RNA sequencing atlas of mechanical strain‐related AS enthesitis. In this context, a disease‐specific subpopulation of SDC1^+^ sheath fibroblasts was identified to arise under mechanical strain, and these cells secreted higher levels of CXCL5 to recruit and promote the activation of CXCR4^hi^ neutrophils, which exacerbated CXCR4^hi^ neutrophil‐mediated enthesitis by increasing their neutrophil extracellular trap formation. Administering CXCL5 neutralizing antibody relieved disease progression in SKG mice. Additionally, computational trajectory analysis revealed a distinct fate branch of the mechanical strain‐responding SDC1^+^ sheath fibroblasts under the control of SOX5‐mediated enhancers and super‐enhancers. Specifically inhibiting SOX5 in enthesis fibroblasts via rAAV9.HAP‐1 carrying a shRNA targeting *Sox5* blocked the generation of SDC1^+^ sheath fibroblasts in response to mechanical strain and markedly reversed the development of CXCR4^hi^ neutrophil‐mediated enthesitis. This study identifies the specific cell interactions and molecular mechanisms involved in mechanical strain‐related AS enthesitis, therefore contributing to the understanding of AS pathogenesis and providing insight into potential clinical treatments for AS.

## Graphical abstract

In ankylosing spondylitis (AS), mechanical strain‐programmed SDC1+ sheath fibroblasts secrete CXCL5 to recruit neutrophils and promote the activation of CXCR4hi neutrophils, thereby exacerbating enthesitis by promoting neutrophil extracellular traps (NETs) formation. SOX5 controls the generation of SDC1+ sheath fibroblasts via super‐enhancers. Both CXCL5 neutralization and SOX5 inhibition in fibroblasts improved mechanical strain‐mediated enthesitis in AS.

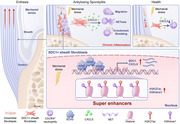

## 1 Introduction

Ankylosing spondylitis (AS) is a common type of osteoimmune disease, with a prevalence ranging from 0.3% to 1.4% [1]. AS, a subtype of axial spondyloarthritis (SpA), manifests as chronic low back pain and new bone formation, leading to spine ankylosis and disability in patients in the later stage of the disease [2]. Although novel therapies, including biological disease‐modifying antirheumatic drugs (bDMARDs), have provided substantial benefit in terms of achieving AS remission in recent years, clinical response rates need further improvement [3]. Elucidating the pathogenesis of AS and identifying critical disease‐causing cell types will contribute greatly to the development of new treatments for AS.

Enthesitis is a pathognomonic feature of AS and psoriatic arthritis that distinguishes them from other osteoimmune diseases [4]. Recent studies have highlighted the critical role of mechanotransduction in immune regulation and inflammatory processes [5]. Specifically, mechanical strain strongly drives the occurrence and development of enthesitis, resulting in the subsequent formation of syndesmophytes in the context of AS [6]. As the major cells involved in mechanotransduction, fibroblasts can transform into disease‐specific subtypes [7, 8] and interact with recruited immune cells under the action of repetitive biomechanical forces, therefore jointly causing enthesitis [9, 10]. However, a single‐cell atlas of AS‐associated enthesitis and a detailed understanding of the interaction between fibroblasts and immune cells in the local microenvironment are still lacking.

Emerging evidence has shown that disease‐specific fibroblast subpopulations aggravate local inflammation by recruiting and regulating the functions of neutrophils [11, 12]. In addition, the critical role of neutrophils in AS‐associated enthesitis has been widely recognized [13]. Previous studies have divided neutrophils into various subtypes, among which neutrophils with increased CXCR4 expression (CXCR4^hi^ neutrophils) are strongly involved in heightened inflammatory responses by increasing the formation of neutrophil extracellular traps (NETs) [14, 15]. Our recent study revealed the pathological effect of NETs in AS [16], but the detailed role of CXCR4^hi^ neutrophils as well as their interaction with fibroblasts in AS enthesitis remain unclear.

In this study, we aimed to generate a single‐cell atlas of mechanical strain‐related enthesitis and identify the specific cell interactions and molecular mechanisms that trigger AS enthesitis. A hind paw loading/unloading model was constructed in SKG mice as an animal model of mechanical strain‐related enthesitis in AS [17, 18]. By single‐cell RNA sequencing (scRNA‐seq) of the enthesis of the Achilles tendon, we generated a single‐cell atlas of enthesitis and then identified mechanical strain‐responding SDC1^+^ sheath fibroblasts. These SDC1^+^ sheath fibroblasts secreted higher levels of CXCL5 to recruit neutrophils and promote the activation of CXCR4^hi^ neutrophils, which then exacerbated CXCR4^hi^ neutrophil‐mediated enthesitis by promoting NETs formation. In addition, the generation of SDC1^+^ sheath fibroblasts was under the control of SOX5 in response to mechanical strain. Administering a CXCL5 neutralizing antibody or specifically inhibiting SOX5 in fibroblasts improved mechanical strain‐mediated enthesitis in AS. This study not only contributes to the understanding of AS pathogenesis but also provides insight into potential clinical treatments for AS.

## 2 Results

### 2.1 Single‐Cell Atlas of Mechanical Strain‐Related Enthesitis in an AS Model

**FIGURE 1:**
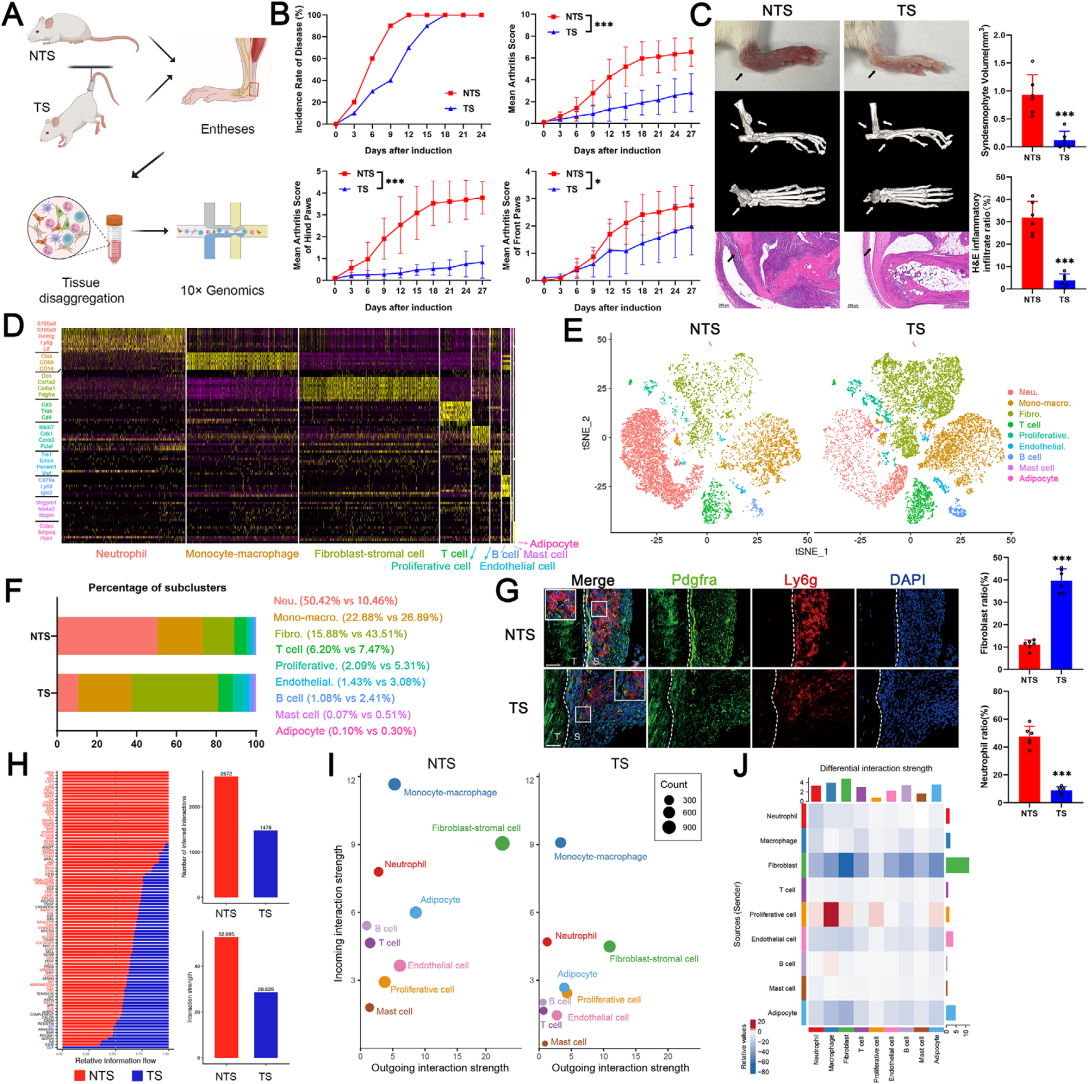
Single‐cell atlas of mechanical strain‐related enthesitis in an ankylosing spondylitis (AS) model. (A) Processing pipeline of single‐cell RNA sequencing of Achilles tendon entheses from SKG mice under nontail suspension (NTS)/tail suspension (TS) conditions. (B) The incidence rate in SKG mice under TS conditions was lower than that in SKG mice under NTS conditions before Day 15. The mean arthritis score, as well as that of the fore paws and hind paws, was lower in the TS group (*n* = 10) than in the NTS group (*n* = 10). (C) The degree of swelling, number of syndesmophytes detected by micro‐CT and severity of inflammatory infiltration detected by HE staining in the entheses were alleviated in the TS group (Black and white arrows). Scale bar, 200 µm. (D) The scaled expression of marker genes for each cluster is shown as a heatmap. (E) T‐distributed stochastic neighbor embedding (t‐SNE) view of 10,312 cells from the NTS group and 13,316 cells from the TS group, color‐coded by the assigned cell type. (F) Proportion of each cell cluster in the NTS and TS groups. (G) The ratio of Ly6g^+^ neutrophils was lower and that of Pdgfra^+^ fibroblasts was greater on the tendon sheath side of the entheses in the TS group (*n* = 6) than in the NTS group (*n* = 6). S indicates the sheath side, and T indicates the tendon side. Scale bar, 50 µm. (H) Relative signal flow in the NTS and TS groups. Both the number and strength of signaling pathway interactions decreased in the TS group. (I) Dot plot showing the strengths of the incoming and outgoing signal interactions of each clustered cell type. (J) Heatmap showing the differential interaction strength of each clustered cell type of the TS groups compared to the NTS groups. The values are presented as the means ± SDs. ^*^ *p* < 0.05 and ^***^ *p* < 0.001. Neu indicates neutrophils, Mono‐macro indicates monocyte–macrophage, Fibro indicates fibroblasts, Proliferative indicates proliferative cells, and Endothelial indicates endothelial cells.

A hind paw loading/unloading model, which is recognized as a well‐established animal model for AS, was constructed in SKG mice, and the enthesis tissue of the Achilles tendon was subsequently disaggregated for scRNA‐seq (Figure 1A). Consistent with previous reports [6, 10], the incidence rate and disease score were both lower in the tail suspension (TS) condition than in the nontail suspension (NTS) condition (Figure 1B). Histological assessment of the hind paws revealed significant swelling and inflammation of the entheses under NTS conditions, and these abnormalities, as well as syndesmophyte formation detected by micro‐CT, were markedly alleviated when the mechanical strain was unloaded under TS conditions on day 27 of induction (Figure 1C). ScRNA‐seq yielded a total of 23,628 cells, including 10,312 cells in the NTS group and 13,316 cells in the TS group. Merged analysis separated these cells into nine distinct clusters on the basis of marker genes: neutrophils (*S100a9*, *S100a8*, *Ly6g*), monocyte‐macrophage (*Ctss*, *Cd68*, *Cd14*), fibroblast‐stromal cells (*Pdgfra*, *Col1a2*, *Col6a1*), T cells (*Cd3*, *Cd4*, *Trac*), proliferative cells (*Mki67*, *Cdk1*), endothelial cells (*Tie1*, *Emcn*, *Pecam1*), B cells (*Cd79a*, *Iglc2*), mast cells (*Mrgprb1*, *Ms4a2*), and adipocytes (*Cidec*, *Adipoq*) (Figure 1D,E and Figure S1). Compared with those in the NTS condition, the relative proportions of fibroblasts increased, but the relative proportions of inflammatory cells, especially neutrophils, decreased in the TS condition (Figure 1E,F). Immunofluorescence staining revealed that the ratio of Ly6g^+^ neutrophils decreased but the ratio of Pdgfra^+^ fibroblasts increased on the tendon sheath side of the enthesis under TS conditions (Figure 1G). Through cell communication analysis, we demonstrated that the number and strength of cellular interactions in the local microenvironment of the enthesis, such as interactions mediated by TNF and WNT signaling, were reduced under TS condition (Figure 1H). Moreover, the outgoing signal interaction strength of fibroblasts was greater than that of other cells, while neutrophils were one of the cells with the greatest incoming signal interaction strength. These signal interactions strength were both weakened under TS conditions (Figure 1I). The differential signal interaction strengths were greatest in fibroblasts of the TS groups compared to the NTS groups (Figure 1J). These results suggested the critical role of fibroblasts and neutrophils in mechanical strain‐related enthesitis.

### 2.2 Subcluster Identification Revealed Mechanical Strain‐Responding SDC1^+^ Sheath Fibroblasts

**FIGURE 2:**
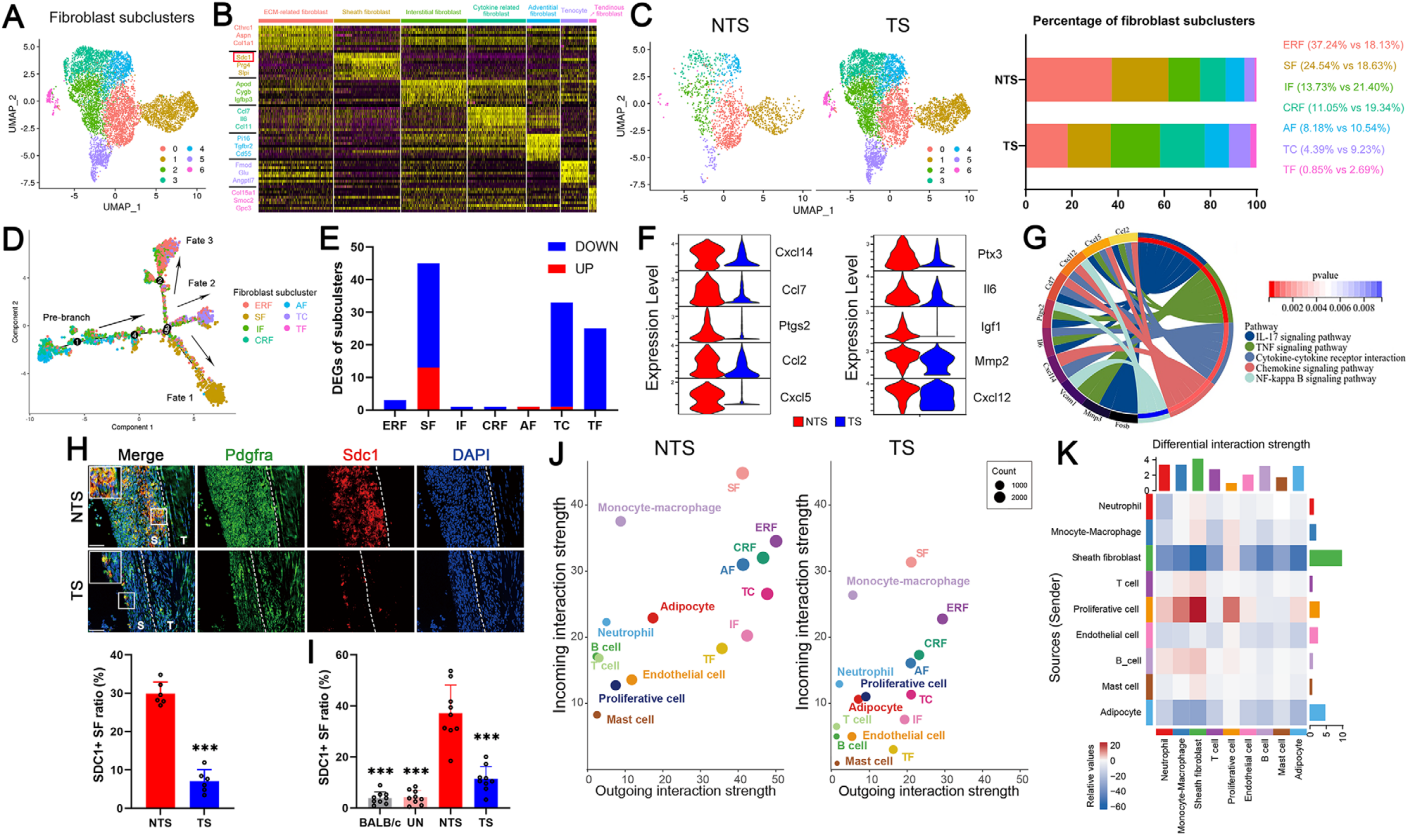
Subcluster identification revealed mechanical strain‐responding SDC1^+^ sheath fibroblasts. (A) Uniform manifold approximation and projection (UMAP) view of 7432 fibroblasts showing seven annotated cell clusters. (B) Heatmap showing the scaled expression of marker genes for seven subclusters of fibroblasts. (C) UMAP visualization of 1638 fibroblasts in the nontail suspension (NTS) group and 5794 fibroblasts in the tail suspension (TS) group and the proportion of each fibroblast subcluster in the NTS and TS groups. (D) Pseudotime trajectory analysis showing the prebranch and different fates of fibroblast subclusters. (E) The number of differentially expressed genes (DEGs) in each fibroblast subcluster. (F) Violin plot showing lower expression of inflammatory factors in the sheath fibroblasts (SF) subcluster in the TS group than in the NTS group. (G) Chord diagram of the KEGG terms enriched in the differentially expressed genes of SDC1^+^ sheath fibroblasts between the NTS and TS groups. (H) Immunofluorescence staining image showing the number and location of SDC1^+^ sheath fibroblasts. The ratio of SDC1^+^ sheath fibroblasts was lower in the TS group (*n* = 6) than in the NTS group (*n* = 6). S indicates the sheath side, and T indicates the tendon side. Scale bar, 50 µm. (I) Flow cytometry results revealed a lower proportion of SDC1^+^ sheath fibroblasts in the entheses of BALB/c mice, un‐diseased SKG mice and diseased SKG mice in the TS group but a greater number in diseased SKG mice in the NTS group (*n* = 9). (J) Dot plot showing the incoming and outgoing signal interaction strengths of each fibroblast subcluster and other cell types. (K) Heatmap showing the differential interaction strength of each clustered cell type of the TS group compared to the NTS group. The values are presented as the means ± SDs. ^***^ *p* < 0.001. ERF indicates extracellular matrix (ECM)‐related fibroblasts, SF indicates sheath fibroblasts, IF indicates interstitial fibroblasts, CRF indicates cytokine‐related fibroblasts, AF indicates adventitial fibroblasts, TC indicates tenocytes, and TF indicates tendinous fibroblasts.

Subcluster analysis separated the fibroblasts into seven subsets. Extracellular matrix (ECM) related fibroblasts (Subcluster 0) were defined on the basis of increased expression of collagen and tissue repair‐related genes. Subcluster 1 was identified as SDC1^+^ sheath fibroblasts expressing *Sdc1* and *Prg4* [19], and subcluster 2 was identified as peri‐epithelial/interstitial fibroblasts expressing *Apod* and *Gsn* [20]. Cytokine‐related fibroblasts (Subcluster 3) were defined on the basis of the expression of several cytokines, including *Ccls*, *Il6*, and *Ptx3*, whereas adventitial fibroblasts (Subcluster 4) were annotated for the expression of *Pi16* [21]. In addition, subcluster 5, which expressed *Fmod*, was defined as tenocytes [19], and subcluster 6, which expressed *Smoc2*, was defined as tendinous fibroblasts [22] (Figure 2A,B and Figure S2). Although the relative proportion of fibroblasts increased under TS conditions, the proportions of ECM‐related fibroblasts and sheath fibroblasts among total fibroblasts decreased, which indicated a positive correlation between mechanical strain and these two fibroblast subsets (Figure 2C). In addition, the mechanosensitive ion channel *Piezo1* was also expressed mainly in these two fibroblast subsets (Figure S3A). However, pseudotime trajectory analysis revealed distinct cell fates for ECM‐related fibroblasts and sheath fibroblasts (Figure 2D). We selected SDC1^+^ sheath fibroblasts for further study for the following reasons: 1. Most differentially expressed genes (DEGs) were observed in the TS condition (Figure 2E); 2. Decreased expression of a series of inflammatory factors, such as *Ccls*, *Cxcls*, and *Il6*, was observed among the DEGs in the TS condition (Figure 2F); and 3. DEGs enriched in inflammatory pathways, including the TNF and IL‐17 signaling pathways, were identified via KEGG analysis (Figure 2G), and terms related to bone mineralization and ECM organization were identified via GO analysis (Figure S3B–D). Tissue immunofluorescence staining revealed that the quantity of SDC1^+^ fibroblasts, which were located mainly on the tendon sheath side of the enthesis, was lower under TS conditions (Figure 2H). As confirmed by flow cytometry, SDC1^+^ sheath fibroblasts were rare in the entheses of normal BALB/c mice and uninduced SKG mice. Consistent with the scRNA‐seq data, the proportion of SDC1^+^ sheath fibroblasts was markedly increased in the entheses of diseased SKG mice but was reduced after mechanical strain unloading under TS conditions (Figure 2I). Subcluster communication analysis revealed that, compared with other subsets, SDC1^+^ sheath fibroblasts had stronger incoming and outgoing signaling interaction strengths, and these values were reduced under TS conditions (Figure 2J). When mechanical strain was unloaded under TS conditions, the signaling interactions of SDC1^+^ sheath fibroblasts with inflammatory cells, such as neutrophils, were weakened compared to the NTS conditions (Figure 2K). These results indicated the pathogenic role of SDC1^+^ sheath fibroblasts in mechanical strain‐related enthesitis in AS.

### 2.3 Mechanical Strain‐Responding SDC1^+^ Sheath Fibroblasts were Elevated in the Enthesitis of AS Patients

**FIGURE 3:**
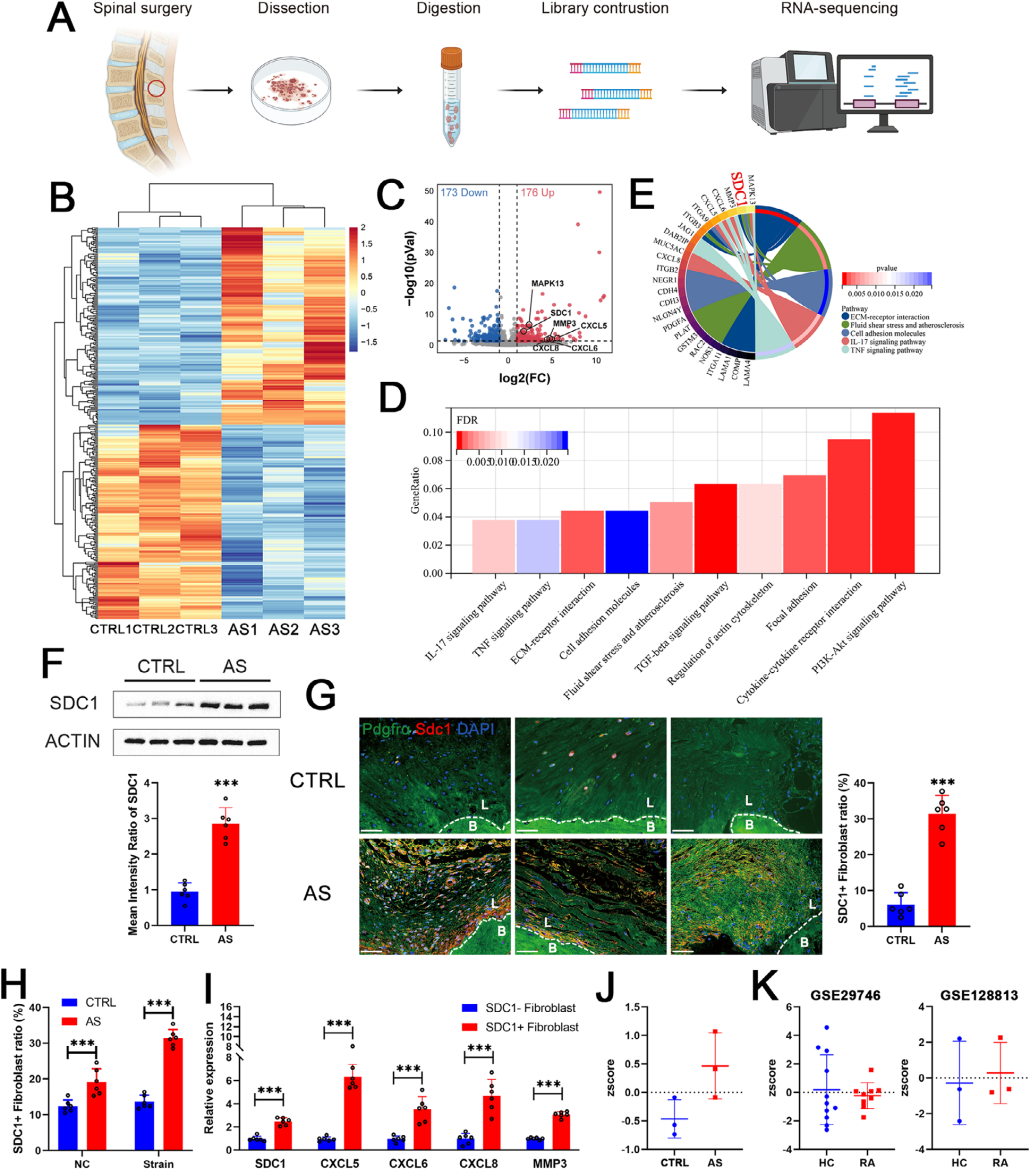
Mechanical strain‐responding SDC1^+^ sheath fibroblasts were increased in enthesitis in ankylosing spondylitis (AS) patients. (A) Processing pipeline of RNA sequencing of fibroblasts isolated from spine entheses. (B) Heatmap showing the differential expression profiles of fibroblasts isolated from the spine entheses of AS patients (AS, *n* = 3) and control patients (CTRL, *n* = 3). (C) Volcano plot showing the upregulated and downregulated genes between the AS and CTRL groups. (D, E) KEGG terms and related differentially expressed genes of fibroblasts isolated from spine entheses of AS patients and controls. (F) Western blotting results showed that SDC1 expression was higher in isolated fibroblasts from AS patients (*n* = 6) than in those from controls (*n* = 6). (G) Immunofluorescence staining image showing the higher ratio of SDC1^+^ fibroblasts in the spine entheses of AS patients (*n* = 6) than in those of controls (*n* = 6). L indicates the spine ligament enthesis, and B indicates bone. Scale bar, 50 µm. (H) Flow cytometry results showed that the SDC1^+^ ratio of fibroblasts was higher in the AS group (*n* = 6) than in the control group (*n* = 6), and this difference widened after strain stimulation. (I) qRT‒PCR results confirmed that SDC1^+^ fibroblasts (*n* = 6) expressed higher levels of *SDC1*, *CXCL5*, *CXCL6*, *CXCL8* and *MMP3* than did SDC1^−^ fibroblasts (*n* = 6) isolated from the spine entheses of AS patients. (J) GSVA results showing a greater z score of the mechanical strain‐responding gene set of SDC1^+^ sheath fibroblasts in the AS group than in the CTRL group. (K) GSVA results showing equal *z* scores of the mechanical strain‐responding gene set of SDC1^+^ sheath fibroblasts in the rheumatoid arthritis (RA) group compared with those in the CTRL group in two other studies. The values are presented as the means ± SDs. ^***^ *p* < 0.001. NTS indicates nontail suspension, and TS indicates tail suspension.

We isolated fibroblasts from the spine entheses of AS patients and control samples for RNA sequencing (Figure 3A). Different expression profiles were observed, and a total of 349 differentially expressed genes, including 173 downregulated and 176 upregulated genes, were identified (Figure 3B,C). These DEGs were enriched in terms such as ECM organization and IL‐17 and TNF‐α signaling through GO and KEGG analyses (Figure 3D,E), which was consistent with the results of the GO and KEGG analyses of mouse SDC1^+^ sheath fibroblasts (Figure 2G and Figure S3B–D). Moreover, SDC1 was identified as one of the DEGs in fibroblasts from the spine entheses of AS patients, which was further confirmed at the protein level (Figure 3E,F). As determined by immunofluorescence staining at the tissue level, many more SDC1^+^ fibroblasts were observed in the spine entheses of AS patients than in those of the control samples (Figure 3G). The flow cytometry results consistently revealed that the SDC1^+^ ratio was greater in fibroblasts isolated from the spine entheses of AS patients. Besides, this ratio significantly increased under tension conditions in AS fibroblasts rather than the control conditions (Figure 3H). The qRT‒PCR results showed that SDC1^+^ fibroblasts from the spine entheses of AS patients expressed increased levels of CXCLs, including CXCL5, confirming the RNA‐seq data of human fibroblasts (Figure 3I). The genes differentially expressed in SDC1^+^ sheath fibroblasts according to scRNA‐seq were defined as “mechanical strain‐responding gene set of AS”. We performed GSVA analysis on the fibroblast RNA‐seq data, and the result showed that the z score for this gene set was significantly increased in AS patients compared to control samples (Figure 3J). To further evaluate the disease‐specific role of SDC1^+^ sheath fibroblasts in AS, we analyzed the *z* scores of the gene sets of in two other studies involving fibroblasts from patients with rheumatoid arthritis (RA) [23, 24]. However, the *z* scores were equal between the RA group and the healthy control group (Figure 3K). These results confirmed the disease‐specific role of mechanical strain‐responding SDC1^+^ sheath fibroblasts to AS enthesitis.

### 2.4 CXCR4^hi^ Neutrophils Mediated Mechanical Strain‐Related Enthesitis in AS

**FIGURE 4:**
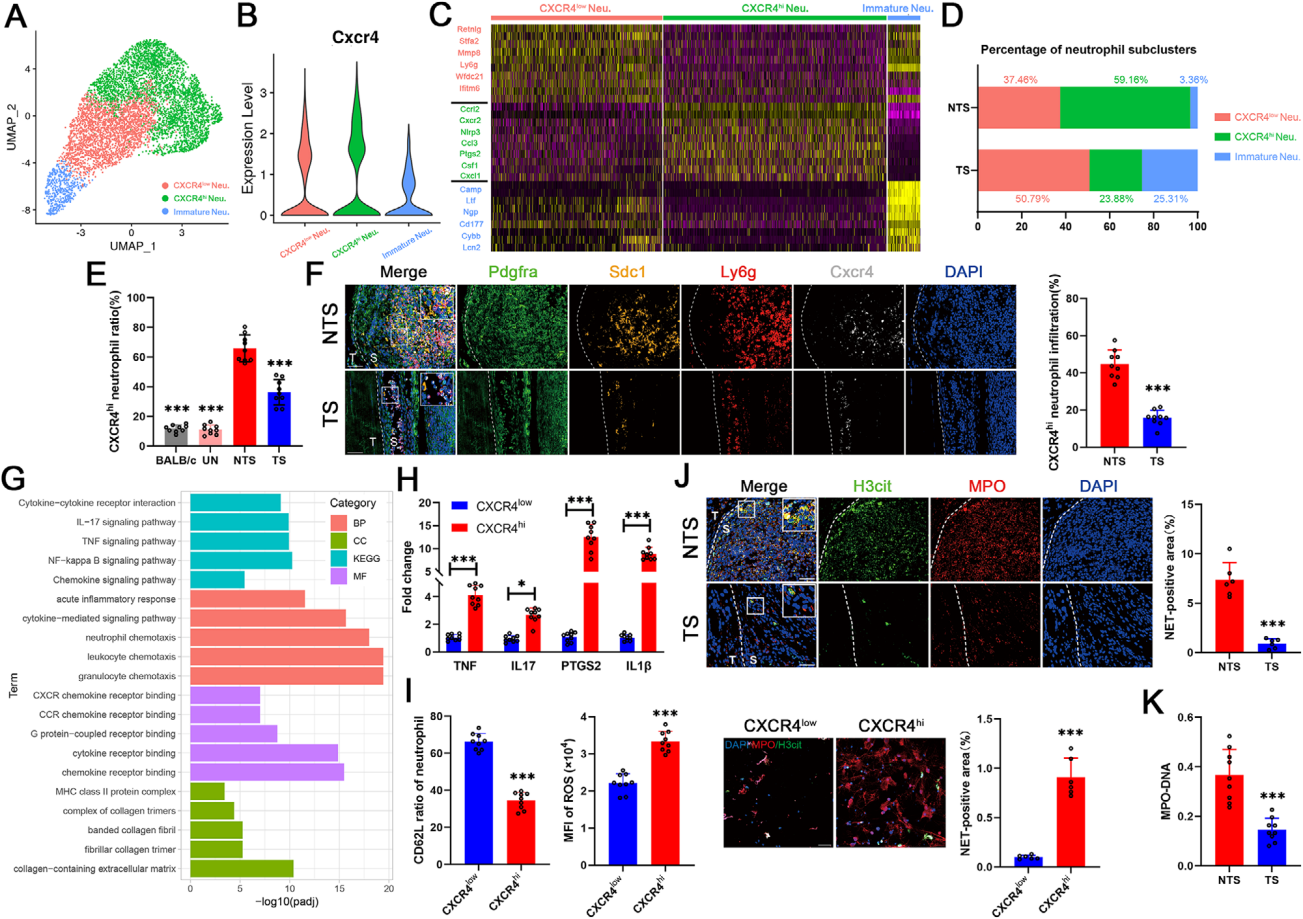
CXCR4^hi^ neutrophils mediated mechanical strain‐related enthesitis in AS. (A) Uniform manifold approximation and projection (UMAP) visualization of 6426 neutrophils showing three annotated cell clusters. (B) Violin plot showing the *Cxcr4* expression of the three neutrophil subpopulations. (C) Heatmap showing the scaled expression of marker genes for three subclusters of neutrophils. (D) Proportion of each neutrophil subcluster in the nontail suspension (NTS) and tail suspension (TS) groups. (E) Flow cytometry plot showing a lower proportion of CXCR4^hi^ neutrophils in the entheses of BALB/c mice, un‐diseased SKG mice and diseased SKG mice in the TS group but a greater number of CXCR4^hi^ neutrophils in diseased SKG mice in the NTS group (*n* = 9). (F) Immunofluorescence staining images showing the number and location of SDC1^+^ sheath fibroblasts and CXCR4^hi^ neutrophils, indicating a lower infiltrative ratio of CXCR4^hi^ neutrophils in the TS group (*n* = 9) than in the NTS group (*n* = 9). S indicates the sheath side, and T indicates the tendon side. Scale bar, 50 µm. (G) Comparison of the GO and KEGG terms enriched in the marker genes of CXCR4^hi^ neutrophils in the NTS and TS groups. (H) qRT‒PCR results showing higher expression levels of *Tnf‐α*, *Il17*, *Ptgs2* and *Il1β* in CXCR4^hi^ neutrophils (*n* = 9) than in CXCR4^low^ neutrophils (*n* = 9). (I) The ratio of CD62L was lower and the ROS level was greater in CXCR4^hi^ neutrophils (*n* = 9) than in CXCR4^low^ neutrophils (*n* = 9). Immunofluorescence staining images showing enhanced NETs (positive for H3cit and MPO) levels in the CXCR4^hi^ neutrophils compared with CXCR4^low^ neutrophils (*n* = 6). Scale bar, 50 µm. (J) Immunofluorescence staining images showing decreased NETs (positive for H3cit and MPO) levels in the enthesitis of SKG mice of the TS group compared with that in the NTS group (*n* = 6). S indicates the sheath side, and T indicates the tendon side. Scale bar, 50 µm. (K) MPO‐DNA levels in the peripheral blood were lower in the TS group (*n* = 9) than in the NTS group (*n* = 9). The values are presented as the means ± SDs. ^*^ *p* < 0.05, ^***^ *p* < 0.001.

Neutrophil subcluster analysis of the scRNA‐seq data was performed to evaluate their status and functions in mechanical strain‐related enthesitis. Similar to previously reported [14, 15], neutrophils were divided into three subclusters based on the CXCR4 expression level: the CXCR4^low^ subpopulation expressing *Retnlg* and *Mmp8*, the CXCR4^hi^ subpopulation expressing various types of inflammatory cytokines, including *Ptgs2*, and the immature subpopulation expressing *Camp* and *Ltf* rather than *Cxcr4* (Figure 4A–C). Among these three subclusters, the proportion of CXCR4^hi^ neutrophils at the enthesis decreased after tail suspension (Figure 4D). As confirmed by flow cytometry, the proportion of CXCR4^hi^ neutrophils at the enthesis increased after disease onset but decreased after mechanical strain unloading in SKG mice (Figure 4E). With respect to spatial location, immunofluorescence staining revealed that CXCR4^hi^ neutrophils were located adjacent to SDC1^+^ sheath fibroblasts on the sheath side of the enthesis and that the number of infiltrating CXCR4^hi^ neutrophils markedly decreased under TS conditions (Figure 4F). GO and KEGG analyses revealed that the marker genes of CXCR4^hi^ neutrophils were enriched in relation to cytokines and inflammation, including IL‐17 and TNF‐α signaling (Figure 4G). We then isolated CXCR4^hi^ and CXCR4^low^ neutrophils from the enthesitis of diseased SKG mice. The results showed that the expression levels of IL‐17, TNF‐α and PTGS2 were increased in CXCR4^hi^ neutrophils (Figure 4H). In addition, compared with CXCR4^low^ neutrophils, CXCR4^hi^ neutrophils had a lower CD62L ratio, elevated ROS levels and enhanced NETs formation (Figure 4I). A large amount of NET formation was observed in the enthesitis of diseased SKG mice, which was significantly decreased after tail suspension (Figure 4J). Moreover, the level of MPO‐bound DNA, another biomarker of NET formation in the peripheral blood, was also reduced in SKG mice under NTS conditions (Figure 4K). These results suggested that CXCR4^hi^ neutrophils contributed greatly to mechanical strain‐related enthesitis in AS.

### 2.5 SDC1^+^ Sheath Fibroblasts Recruited and Regulated the Function of CXCR4^hi^ Neutrophils by Secreting CXCL5

**FIGURE 5:**
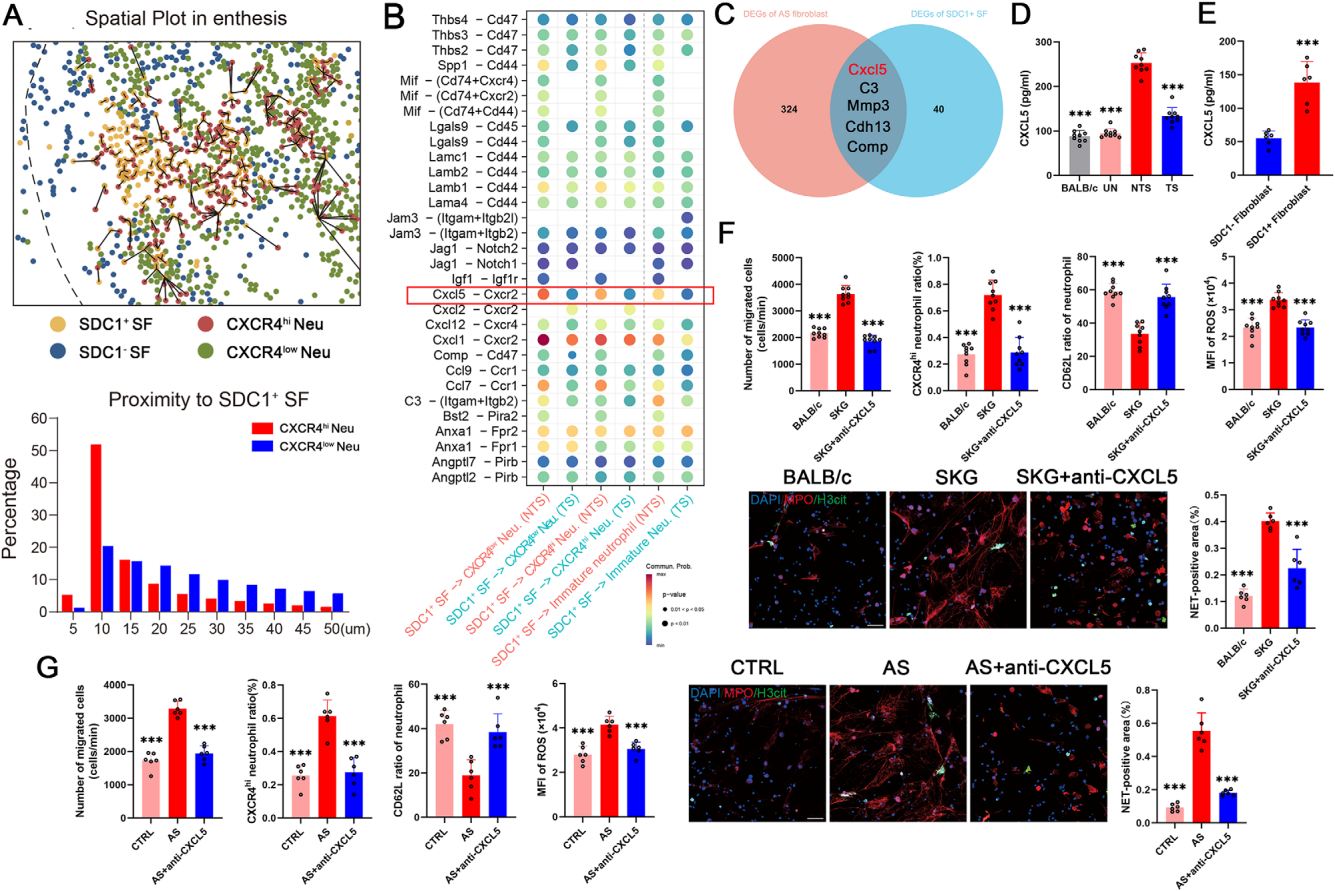
SDC1^+^ sheath fibroblasts recruited and regulated the function of CXCR4^hi^ neutrophils by secreting CXCL5. (A) Spatial dot plot showing the cellular distances of CXCR4^hi^ and CXCR4^low^ neutrophils to SDC1^+^ fibroblasts. (B) Dot plot showing decreased signaling in the TS group. The color of the dots indicates the communication probability (strength) of a certain signaling pair between different cell types. The size of the dots represents the P value of a certain signaling pair between different cell types. (C) Venn diagram showing five common differentially expressed genes (DEGs) identified via RNA‐seq of human fibroblasts and scRNA‐seq of SKG fibroblasts. (D) ELISA results showing lower CXCL5 levels in fibroblasts from BALB/c mice, un‐diseased SKG mice and diseased SKG mice in the TS group but higher CXCL5 levels in fibroblasts from diseased SKG mice in the nontail suspension (NTS) group (*n* = 9). (E) ELISA results showing that higher secretion level of CXCL5 in SDC1^+^ fibroblasts (*n* = 6) than in SDC1^−^ fibroblasts (*n* = 6) isolated from AS spine entheses. (F) Compared with fibroblasts from BALB/c mice, fibroblasts from diseased SKG mice showed enhanced abilities to recruit mice neutrophils, increase the CXCR4^hi^ neutrophils ratio, ROS level and NETs formation (positive for H3cit and MPO) and decrease the CD62L ratio; these effects were eliminated by a mouse CXCL5 neutralizing antibody (*n* = 9). Scale bar, 50 µm. (G) Compared with control fibroblasts, fibroblasts from AS patients showed enhanced abilities to recruit human neutrophils, increase the CXCR4^hi^ neutrophils ratio, ROS level and NETs formation (positive for H3cit and MPO) and decrease the CD62L ratio; these effects were eliminated by a human CXCL5 neutralizing antibody (*n* = 6). Scale bar, 50 µm. The values are presented as the means ± SDs. ^***^ *p* < 0.001.

We then analyzed the proximity of neutrophils to SDC1^+^ sheath fibroblasts in a spatial plot of enthesitis and determined that SDC1^+^ sheath fibroblasts had a shorter spatial distance to CXCR4^hi^ neutrophils than to CXCR4^low^ neutrophils (Figure 5A). Cell communication analysis revealed that SDC1^+^ sheath fibroblasts interacted with CXCR4^hi^ neutrophils through various cytokine signals, including CXCL5, in the context of mechanical strain‐related enthesitis, which was markedly weakened after tail suspension (Figure 5B). Among these cytokine signals, CXCL5 was collectively the DEG with higher expression in RNA‐seq data from fibroblasts isolated from the spine enthesitis of AS patients and in scRNA‐seq from SDC1^+^ sheath fibroblasts of SKG mice (Figure 5C). Besides, the CXCL5 concentration in entheses was elevated after disease onset but decreased after mechanical strain unloading in SKG mice (Figure 5D) and was also higher in SDC1^+^ fibroblasts than in SDC1^−^ fibroblasts from spine enthesitis of AS patients (Figure 5E). Fibroblasts from diseased SKG mice and spine enthesitis of AS patients showed similar potential to promote mice or human neutrophil recruitment and increase the CXCR4^hi^ neutrophils ratio. Besides, both the NETs formation and the activation level determined by ROS level and CD62L ratio of neutrophils were elevated when cultured with enthesitis fibroblast from diseased SKG mice or AS patients. These effects were reversed by the CXCL5 neutralizing antibodies (Figure 5F,G). Adding exogenous CXCL5 resulted in similar abilities to recruit and activate the functions of neutrophils (Figure S4A,B). Administering a CXCL5 neutralizing antibody reduced the arthritis score of SKG mice and relieved their inflammatory infiltration and syndesmophyte formation (Figure S4C,D). As shown by immunofluorescence staining and flow cytometry, the CXCL5 neutralizing antibody significantly decreased the infiltrative ratio of CXCR4^hi^ neutrophils on the sheath side of the enthesis (Figure S4E,F). Additionally, administration of a CXCL5 neutralizing antibody decreased NET formation in the enthesitis and MPO‐bound DNA in the peripheral blood of SKG mice (Figure S4G,H). These results demonstrated that SDC1^+^ sheath fibroblasts recruited and regulated the function of CXCR4^hi^ neutrophils through CXCL5.

### 2.6 Mechanical Strain Programmed SDC1^+^ Sheath Fibroblast Generation through SOX5

**FIGURE 6:**
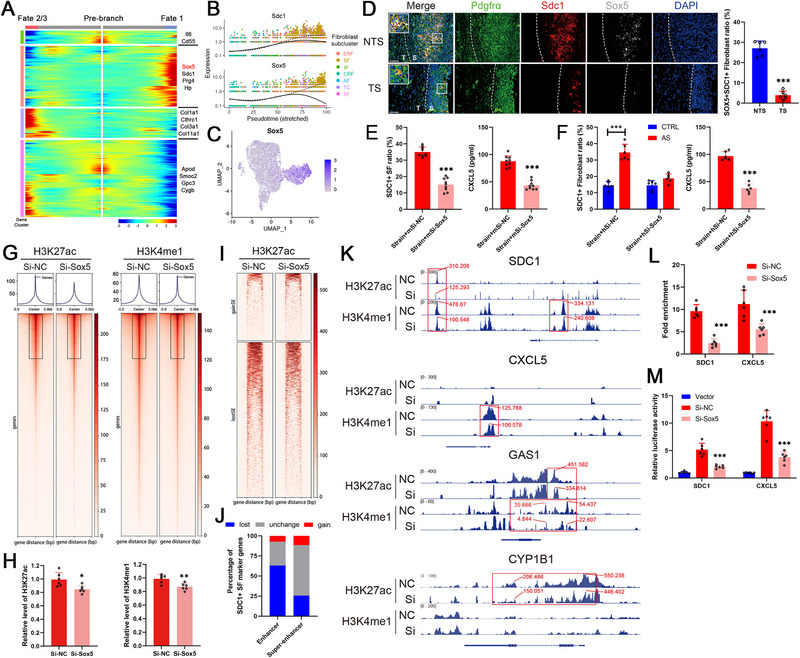
Mechanical strain programmed SDC1^+^ sheath fibroblast generation through SOX5. (A) Heatmap showing the scaled expression of differentially expressed genes related to fibroblast fates according to pseudotime analysis, cataloged into four major gene clusters (labels on right). (B) Pseudotime kinetics of *Sdc1* and *Sox5* from the root of the trajectory to fate 2/3 (solid line) and the cells up to fate 1 (dashed line). ERF indicates extracellular matrix (ECM)‐related fibroblasts, SF indicates sheath fibroblasts, IF indicates interstitial fibroblasts, CRF indicates cytokine‐related fibroblasts, AF indicates adventitial fibroblasts, TC indicates tenocytes, and TF indicates tendinous fibroblasts. (C) Uniform manifold approximation and projection (UMAP) visualization color‐coded according to the expression (gray to purple) of *Sox5*. (D) Immunofluorescence staining showing the ratio and location of SDC1^+^SOX5^+^ sheath fibroblasts (*n* = 6). S indicates the sheath side, and T indicates the tendon side. Scale bar, 50 µm. (E) Decreased SDC1^+^ ratio of fibroblasts from SKG mice and reduced CXCL5 secretion after mSi‐*Sox5* transfection under strain conditions (*n* = 9). (F) Decreased SDC1^+^ ratio of fibroblasts from AS patients and their reduced CXCL5 secretion after hSi‐*Sox5* transfection under strain conditions (*n* = 6). (G) Heatmap showing H3K27ac and H3K4me1 levels in fibroblasts transfected with Si‐NC or Si‐*Sox5*. (H) The quantitative analysis of H3K27ac and H3K4me1 levels in fibroblasts transfected with Si‐NC or Si‐*Sox5*. (I) Heatmap of superenhancers (SEs) identified by H3K27ac in fibroblasts transfected with Si‐NC or Si‐*Sox5*. (J) Histogram showing the proportion of SDC1^+^ sheath fibroblast marker genes whose enhancer/SE signal changed. (K) H3K27ac and H3K4me1 signal traces of *Sdc1*, *Cxcl5*, *Gas1* and *Cyp1b1*, and red boxes showed the reduced signals. (L) High enrichment signals of the enhancer regions of *Sdc1* and *Cxcl5* were significantly reduced after Si‐*Sox5* transfection (*n* = 6). (M) The enhancer of *Sdc1* and *Cxcl5* increased luciferase activity, which was markedly suppressed after Si‐*Sox5* transfection (*n* = 6). The values are presented as the means ± SDs. ^***^ *p* < 0.001.

By scanning the node genes to determine the fates of fibroblasts via pseudotime trajectory analysis, we found that the expression profile of the transcription factor *Sox5* was similar to that of *Sdc1*, which were both highly expressed in the sheath fibroblast subpopulation (Figure 6A–C). In addition, SOX5 and SDC1 colocalized in fibroblasts at the enthesis, and the number of SDC1^+^SOX5^+^ sheath fibroblasts decreased under TS conditions (Figure 6D). To evaluate the effect of SOX5, we constructed specific siRNAs for use in mouse or human fibroblasts and verified their inhibitory efficiency (Figure S5). The proportion of SDC1^+^ fibroblasts isolated from SKG mice, as well as their CXCL5 secretion level, was decreased by transfection of SOX5 siRNA under tension application in vitro (Figure 6E). Moreover, the proportion of SDC1^+^ fibroblasts in AS patients was much greater than that in the control group under tension conditions, and this difference as well as the increased CXCL5 secretion level were also reversed after SOX5 inhibition (Figure 6F). H3K27ac and H3K4me1 modifications can activate enhancer elements or even form superenhancers (SEs) to promote the expression of specific genes, and this process has been reported to be modulated by SOX5 [25]. In fibroblasts, the signal intensity of both H3K27ac and H3K4me1 modifications was attenuated after transfection with SOX5 siRNA (Figure 6G). Consistently, the quantitative analysis using EpiQuik Global Acetyl Histone H3K27 Quantification Kit and EpiQuik Global Pan‐Methyl Histone H3K4 Quantification Kit showed that the levels of H3K27ac and H3K4me1 were lower in the si‐SOX5 group (Figure 6H). In addition, a total of 2034 decreased SEs and 1215 lost SEs in the si‐SOX5 group were identified on the basis of the H3K27ac modification level (Figure 6I). SOX5 knockdown resulted in the loss or reduction of enhancer signals for approximately 63% of the marker genes of SDC1^+^ sheath fibroblast, and the loss or reduction of super‐enhancer signals for 25% of them, indicating a critical role of SOX5 in regulating the generation of SDC1^+^ sheath fibroblasts by governing the formation of enhancers/super‐enhancers (Figure 6J). The H3K27ac and H3K4me1 modification levels of marker genes such as *Sdc1*, *Cxcl5*, *Gas1* and *Cyp1b1* were decreased after si‐SOX5 transfection (Figure 6K). Similarly, the H3K27ac modification levels of these marker genes in fibroblasts from AS patients were greater than those in control fibroblasts under strain conditions, and this effect was also eliminated by si‐SOX5 transfection (Figure S6). SOX5‐specific CUT&Tag‐qPCR assay showed that the high enrichment signals of the enhancer regions of *Sdc1* and *Cxcl5*, and knocking down *Sox5* significantly reduced the enrichment signals in these enhancer regions (Figure 6L). Moreover, the enhancer of *Sdc1* and *Cxcl5* increased luciferase activity, which was markedly suppressed after si‐SOX5 transfection (Figure 6M). These results suggested that the generation of mechanical strain‐responding SDC1^+^ sheath fibroblasts is under the control of SOX5 in AS, and that SOX5 directly bound to the enhancer regions of *Sdc1* and *Cxcl5* to regulated their transcriptional activities.

### 2.7 Specific Inhibition of SOX5 Blocked the Generation of Mechanical Strain‐Responding SDC1^+^ Sheath Fibroblasts and CXCR4^hi^ Neutrophil‐Mediated Development of Enthesitis

**FIGURE 7:**
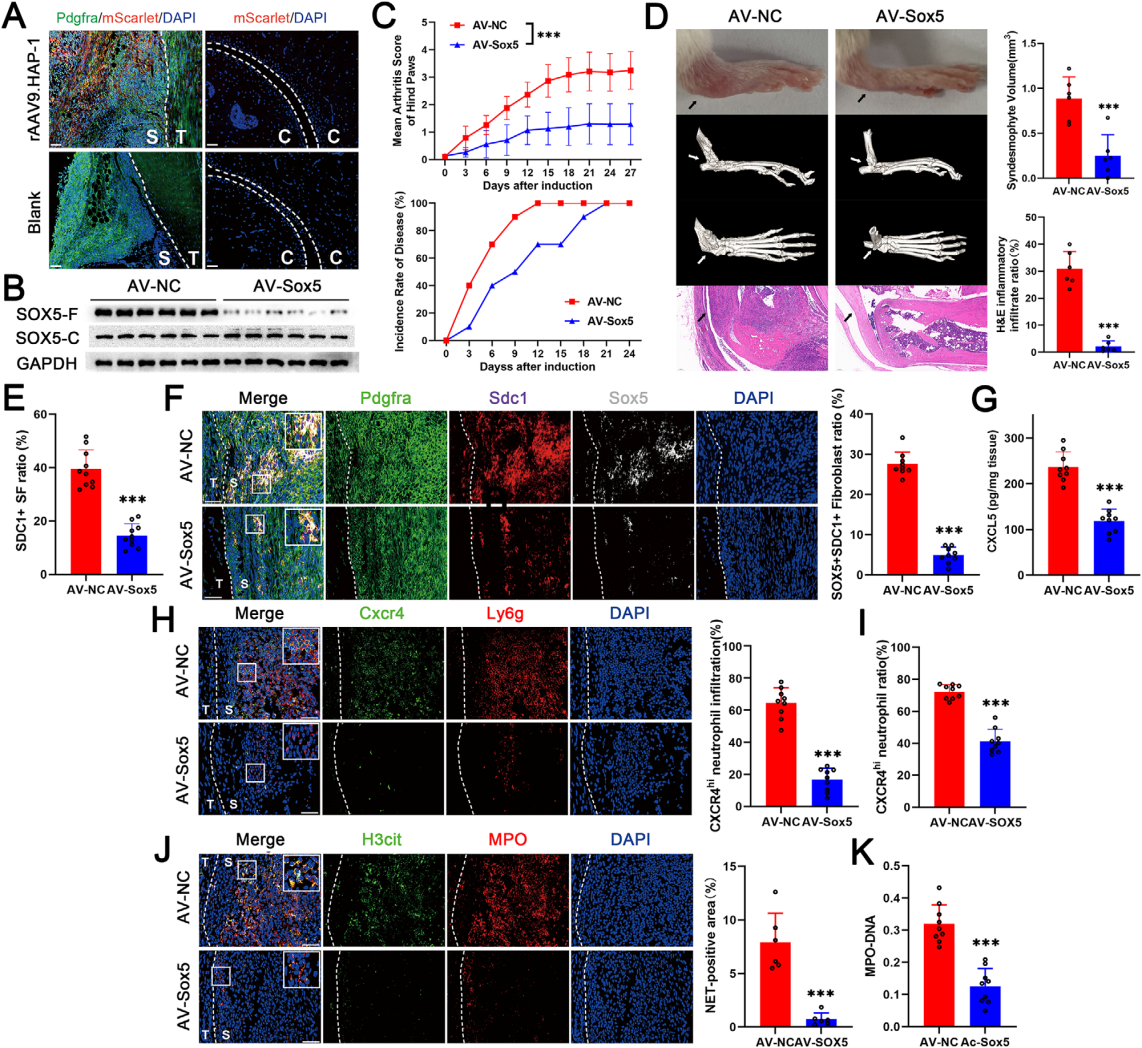
Specific inhibition of SOX5 blocked the generation of mechanical strain‐responding SDC1^+^ sheath fibroblasts and CXCR4^hi^ neutrophil‐mediated development of enthesitis. (A) Immunofluorescence staining image showing rAAV9.HAP‐1 transfection efficiency and specificity in fibroblasts. (B) Western blot results showing decreased SOX5 expression in fibroblasts isolated from AV‐SOX5‐treated SKG mice compared with those from AV‐NC‐treated SKG mice. (C) The ankylosing spondylitis (AS) incidence rate of SKG mice in the AV‐SOX5 group was lower than that of the mice in the AV‐NC group before Day 18. The mean arthritis score was lower in the AV‐SOX5 group (*n* = 10) than in the AV‐NC group (*n* = 10). (D) Compared with those in the AV‐NC group, the degree of swelling, the number of syndesmophytes and the severity of inflammatory infiltration at the entheses were alleviated in the AV‐SOX5 group on day 27 of induction (Black and white arrows). Scale bar, 200 µm. (E) Flow cytometry results showing a decreased SDC1^+^ ratio of fibroblasts in AV‐SOX5‐treated SKG mice (*n* = 10) compared with those in the AV‐NC group (*n* = 10). (F) Immunofluorescence staining image showing a lower ratio of SOX5^+^SDC1^+^ sheath fibroblasts in the entheses of AV‐SOX5‐treated SKG mice (*n* = 9) than in those of AV‐NC‐treated SKG mice (*n* = 9). (G) ELISA results showing lower CXCL5 levels in the entheses of AV‐SOX5‐treated SKG mice (*n* = 9) than in those of AV‐NC‐treated SKG mice (*n* = 9). (H) Immunofluorescence staining image showing a lower ratio of CXCR4^hi^ neutrophils in the entheses of AV‐SOX5‐treated SKG mice (*n* = 9) than in those of AV‐NC‐treated SKG mice (*n* = 9). (I) Flow cytometry results showing a decreased ratio of CXCR4^hi^ neutrophils in AV‐SOX5‐treated SKG mice (*n* = 9) compared with those in the AV‐NC group (*n* = 9). (J) Immunofluorescence staining image showing decreased NETs (positive for H3cit and MPO) levels in the entheses of AV‐SOX5‐treated SKG mice compared with those of AV‐NC‐treated SKG mice (*n* = 6). (K) MPO‐DNA levels in the peripheral blood were lower in the AV‐SOX5 group (*n* = 9) than in the AV‐NC group (*n* = 9). The values are presented as the means ± SDs. ^*^ *p* < 0.05, ^***^ *p* < 0.001. S indicates the tendon sheath, T indicates the tendon, and C indicates the articular cartilage. Scale bar of the immunofluorescence staining image, 50 µm.

As previously reported [26], we constructed a fibroblast‐targeting adeno‐associated virus (AAV) vector (rAAV9.HAP‐1) carrying a shRNA sequence targeting *Sox5* (AV‐SOX5). After local injection into the entheses of SKG mice, rAAV9.HAP‐1 exhibited fibroblast specificity and did not affect other cells, such as chondrocytes (Figure 7A). To confirm the interference efficiency, fibroblasts and chondrocytes were isolated. The SOX5 protein expression of fibroblast (SOX5‐F) in the AV‐SOX5 group was found to be lower than that in the AV‐NC group, but no difference was observed in the SOX5 protein expression of chondrocytes (SOX5‐C) between the two groups (Figure 7B). Both the disease score and incidence rate were reduced in AV‐SOX5‐injected SKG mice, and inflammatory infiltration and syndesmophyte formation were also attenuated (Figure 7C,D). As determined by flow cytometry, the proportion of SDC1^+^ sheath fibroblasts was markedly reduced in the AV‐SOX5 group (Figure 7E). Similarly, SOX5 expression in fibroblasts decreased after AV‐SOX5 injection, and the number of SOX5^+^SDC1^+^ fibroblasts also decreased in vivo (Figure 7F). In addition, the CXCL5 level of the enthesitis was reduced in the AV‐SOX5 group (Figure 7G). As shown by immunofluorescence staining and flow cytometry, the infiltration ratio of CXCR4^hi^ neutrophils on the sheath side of the enthesis decreased after AV‐SOX5 treatment (Figure 7H,I), accompanied by decreased NET formation in the enthesitis and MPO‐bound DNA in the peripheral blood (Figure 7J,K). Besides, the SDC1^+^ sheath fibroblasts ratio, the CXCR4^hi^ neutrophils ratio and the SOX5 expression levels of fibroblasts were all positively correlated to the mean arthritis score of diseased SKG mice (Figure S7). These results determined that specifically inhibiting SOX5 blocked the generation of mechanical strain‐responding SDC1^+^ sheath fibroblasts and the subsequent CXCR4^hi^ neutrophil‐mediated development of enthesitis in AS.

## 3 Discussion

AS is a common type of osteoimmune disease that leads to spine ankylosis and disability in patients [1]. With respect to pathognomonic features, AS and psoriatic arthritis, two subtypes of SpA, are distinguished from other osteoimmune diseases by the presence of enthesitis [4]. The term enthesis describes the site of insertion of tendons and ligaments into the bone; the enthesis transduces mechanical forces from the muscle to the skeletal system [27]. Even in standing or sitting positions at rest, the enthesis of the axial skeleton such as sacroiliac joint still sustain the high mechanical stress loading to maintain the body posture [28]. In healthy individuals, enthesitis of a certain muscle is common under mechanical overload conditions, such as excessive sports activity. The difference in AS is that enthesitis involves a variety of entheses at the same time and shows chronic characteristics [29], indicating the participation of different pathogenic cells and molecular mechanisms. A previous clinical study demonstrated that AS patients with a history of jobs requiring dynamic flexibility had greater functional limitations and radiographic damage [30]. Additionally, hind paw unloading via tail suspension reduces the enthesitis score and inhibits new bone formation in AS mice [6]. These results confirm the critical effect of mechanical strain on AS‐associated enthesitis, but the detailed changes in the local microenvironment resulting from mechanical strain remain unclear.

Previous studies concerning enthesitis in AS focused on a single cell type [31, 32, 33], and scRNA‐seq overcomes this limitation by exploring the tissue microenvironment and cell interactions. In this study, we constructed a hind paw loading/unloading model in SKG mice, as previously reported [6, 10], to evaluate the effects of mechanical strain and then isolated the enthesis of Achilles tendons for scRNA‐seq. As the incidence rate and disease score improved, the proportion of fibroblasts increased, but the proportion of neutrophils decreased in the cell atlas of the enthesis, as shown by scRNA‐seq and then confirmed by tissue immunofluorescence staining. Similar to the findings of a recent scRNA‐seq employed to evaluate the spinal ligament entheses from AS patients [34], these results indicated the critical role of fibroblasts and neutrophils in mechanical strain‐related enthesitis in AS. Nevertheless, the opinion that mechanical strain triggers enthesitis by directly activating resident immune cells predominates in the field of AS research [35, 36, 37]. Fibroblasts account for the largest proportion of cells in the enthesis, and are the major cells involved in mechanotransduction [7]. In our study, we found that fibroblasts were the cells with the strongest outgoing signal strength at the enthesis and that other cells, including neutrophils, were instead passive signal‐receiving cells. In addition, the ability of fibroblasts to deliver various signals to other cells was markedly inhibited by mechanical strain unloading. We suggest that fibroblasts, rather than immune cells, function as mechanotransductive signal converters in AS enthesitis, sensing and transforming the physical signal of mechanical strain into chemical signals such as cytokines to communicate with other cells in the local microenvironment.

Owing to their high degree of heterogeneity, fibroblasts can turn into diverse subtypes under different conditions, acquiring distinct abilities and ultimately participating in the development of various diseases [8]. Previous studies have reported the effects of fibroblasts on AS pathogenesis [38, 39, 40], but the specific subclusters responsible for AS‐associated enthesitis remain unclear. Through subcluster analysis of our scRNA‐seq data, we demonstrated that SDC1^+^ fibroblasts located on the sheath side of the enthesis were positively related to disease progression, as their proportion increased after disease onset but decreased after disease remission. Moreover, SDC1^+^ sheath fibroblasts were also positively related to mechanical strain, as indicated by their higher *Piezo1* expression, reduced proportion and decreased incoming/outcoming signaling interaction strength after mechanical strain unloading. Additionally, SDC1^+^ sheath fibroblasts may contribute to mechanical strain‐related inflammation in AS entheses, as their DEGs, including those encoding various inflammatory cytokines induced by mechanical strain, were enriched in several AS‐related terms, such as TNF and IL17 signaling. Therefore, we determined that SDC1^+^ sheath fibroblasts constitute the major subset related to the mechanical strain response in the context of AS‐associated enthesitis.

Lining synovial fibroblasts characteristically expressing *Prg4*, another marker gene of sheath fibroblasts according to our scRNA‐seq data, correlated positively with the severity of RA [41]. Do the SDC1^+^ sheath fibroblasts identified in this study constitute an AS‐specific pathogenic subpopulation, or are they involved in other inflammatory diseases? We further demonstrated that the SDC1 expression level was higher in fibroblasts isolated from spine enthesitis of AS patients and that the number of SDC1^+^ fibroblasts was greater in the spine entheses of AS patients. Besides, according to the GSVA results, the DEG set score of SDC1^+^ sheath fibroblasts in the scRNA‐seq data was greater in the fibroblasts isolated from the spine entheses of AS patients than in those from the corresponding controls, as determined by the RNA‐seq data. Nevertheless, no difference in the DEG set score was detected between fibroblasts from RA patients and those from controls in two other independent studies [23, 24]. These data suggest that SDC1^+^ sheath fibroblasts could be AS‐specific pathogenic cells, but this possibility still needs further investigation.

Previous studies revealed that fibroblasts triggered local inflammation by regulating the functions and status of immune cells, therefore leading to arthritis and enthesitis [10, 42]. In this study, we determined that the ratio of neutrophils, rather than other immune cells, significantly decreased under TS conditions, indicating the critical role of neutrophils in mechanical strain‐related enthesitis, as previously reported [13]. Moreover, further subpopulation analysis showed that CXCR4^hi^ neutrophils positively responded to mechanical strain and were related to disease onset. CXCR4^hi^ neutrophils constitute a newly identified subtype with enhanced potential to form NETs and exacerbate local inflammation [14, 15, 43]. Recently, we demonstrated the pathogenic effect of elevated NETs on the development of AS, and targeting NETs has been shown to have a therapeutic effect on AS [16]. In the present study, the systemic and local levels of NETs were reduced under TS conditions, accompanied by a decreased ratio of CXCR4^hi^ neutrophils, revealing the origin of elevated NETs and the importance of CXCR4^hi^ neutrophils in AS‐associated enthesitis.

The status and functions of neutrophils are strongly regulated by fibroblasts in the microenvironment, leading to the progression of inflammatory diseases [44, 45]. As previously reported, neutrophils in the peripheral blood exhibit minimal expression of CXCR4, and the CXCR4^+^ neutrophils were generated under inflammatory stimulation or the effect of other cells in the peripheral blood [46]. Besides, fibroblast‐like synoviocytes expressing SDC1 exacerbated the inflammatory landscape by increasing inflammatory cytokine secretion [47]. Owing to the closer spatial distance shown in our study, we supposed that SDC1^+^ sheath fibroblasts may trigger mechanical strain‐related enthesitis by recruiting and promoting the generation of CXCR4^hi^ neutrophils in the local site. Further investigation determined that CXCL5 was the collective DEG with higher expression in fibroblasts isolated from spine enthesitis of AS patients and SDC1^+^ sheath fibroblasts from SKG mice and that the CXCL5 signaling between SDC1^+^ sheath fibroblasts and CXCR4^hi^ neutrophils was significantly weakened under TS conditions. CXCL5 is a critical cytokine that participates in neutrophil recruitment and NETs formation [48, 49]. In this study, fibroblasts from AS patients or SKG mice showed increased potential to recruit neutrophils and increase the CXCR4^hi^ neutrophil ratio and NETs formation, which were reversed by a CXCL5 neutralizing antibody. Moreover, administration of a CXCL5 neutralizing antibody decreased the CXCR4^hi^ neutrophil ratio and NETs formation level at the enthesis site and relieved disease progression in SKG mice. These data highlighted the role of CXCL5 in the paracrine effect of SDC1^+^ sheath fibroblasts on CXCR4^hi^ neutrophils, as well as in the pathogenesis of AS.

Chronic inflammation and pathological osteogenesis are two pathological changes that occur successively in AS patients [50]. A previous study revealed that sheath fibroblasts are highly involved in heterotopic ossification [51]. Except for the terms related to inflammation, the results of GO and KEGG analyses of the DEGs in SDC1^+^ sheath fibroblasts, fibroblasts isolated from SKG mice and AS patient fibroblasts showed that terms related to osteogenesis, including ossification and ECM‒receptor interactions, were collectively enriched in these three datasets. Besides, syndesmophyte formation was alleviated in SKG mice treated with AV‐SOX5, which the ratio of SDC1^+^ sheath fibroblasts was markedly reduced. We propose that SDC1^+^ sheath fibroblasts may be also involved in the development of AS osteogenesis through two steps: firstly, SDC1^+^ sheath fibroblasts promoted CXCR4^hi^ neutrophils related inflammation, and secondly, they remodeled the ECM after inflammatory destruction to provide a suitable microenvironment conducive to the osteogenic differentiation of stroma cells.

Cell lineage analysis revealed distinct fates of SDC1^+^ sheath fibroblasts, which are under the control of mechanical strain. Through the branched expression analysis modeling (BEAM) of the pseudotime trajectory, we demonstrated that the transcription factor SOX5 was a critical driving molecule for SDC1^+^ sheath fibroblasts responding to mechanical strain. The importance of SOX5 in determining the fate of fibroblasts was previously reported because of its regulatory effect on the emergence of lining synovial fibroblasts in the joints [52, 53]. We further demonstrated that inhibiting SOX5 expression counteracted the ability of mechanical strain to promote SDC1^+^ sheath fibroblast generation both in mice and in humans, suggesting that the decisive role of SOX5 in the fibroblast subpopulation is closely related to mechanical strain. SOX5 promotes the expression of specific genes by activating enhancer elements and forming SEs [25]. Disordered expression of SOX5 leads to fibroblast dysfunction and subsequently contributes to the development of inflammatory diseases such as RA [54, 55]. In this study, SOX5 orchestrated the expression of marker genes of SDC1^+^ sheath fibroblasts through enhancers and SEs, thereby promoting the generation of this subpopulation and the development of inflammation and osteogenesis related to AS. As SOX5 is also a critical regulator for bone and cartilage development [56], whether SOX5 can directly promoted osteogenesis of fibroblast in AS still needs further investigation.

Various novel drugs, including bDMARDs targeting TNF‐α or IL17, have been developed for AS treatment in recent years. However, the clinical response rate (ASAS20) of these treatments, even the most effective one, is approximately 70% [3, 57, 58], suggesting the limitations of therapies that target a single cytokine. On the basis of our results above, we wondered whether specifically inhibiting SOX5 expression to block the generation of mechanical strain responding SDC1^+^ sheath fibroblasts, the upstream converters of mechanical strain signals, would have improved therapeutic effects on AS. Through in vivo experiments, we determined that AV‐SOX5 significantly decreased the proportion of SDC1^+^ sheath fibroblasts, as well as the subsequent ratio of CXCR4^hi^ neutrophils and their NETs formation, in the entheses and markedly inhibited the development of AS‐associated enthesitis. These results provide new insight into therapeutic approaches for AS involving turning off fibroblasts rather than immune cells or cytokines, and further confirmation via clinical research is needed.

In conclusion, our study revealed the central role of the mechanical strain‐responding and SOX5‐programmed SDC1^+^ sheath fibroblasts in triggering CXCR4^hi^ neutrophil‐mediated enthesitis by CXCL5 in AS. These findings provide novel mechanistic insight into the pathogenesis of AS‐associated enthesitis, as well as ideas for therapeutic strategies in the clinic. Several limitations remain. First, although the AS animal models in the current study are well established, mice cannot perfectly model stress in humans. Second, more enthesis tissues from AS patients are needed for scRNA‐seq and spatial transcriptomics. Thirdly, the detailed regulatory mechanisms of SDC1^+^ sheath fibroblasts and other immune cells also need further investigation. Finally, a SOX5‐specific conditional knocking down mouse based on the SKG background could significantly strengthen the conclusion drawn in the present study. These limitations should be addressed via further investigation in the future.

## 4 Materials and Methods

### 4.1 Study Approval

This study was approved by the Ethics Committee of the Eighth Affiliated Hospital, Sun Yat‐Sen University, Guangzhou, China. Six AS patients were enrolled, along with six trauma patients with spinal fractures who required spine surgery and were enrolled as controls. The AS patients met the 1984 Revised New York Diagnostic Criteria for AS, and the trauma patients did not have AS or other rheumatic diseases, infectious diseases or tumors. Informed consent was obtained from all patients before surgery. To minimize the impact of therapy, all patients discontinued treatment using nonsteroidal anti‐inflammatory drugs (NSAIDs), disease modifying antirheumatic drugs (including csDMARDs, bDMARDs, and tDMARDs) and glucocorticoids for 14 days prior to surgery. The experiments involving mice were approved by the Institutional Animal Care and Use Committee of Sun Yat‐Sen University, Guangzhou, China.

### 4.2 Animal Models

SKG mice were purchased from the Shanghai Model Organisms Center, and wild‐type BALB/c mice were purchased from the Laboratory Animal Center of Sun Yat‐Sen University. The mice were housed under a 12‐h light/dark cycle under specific pathogen‐free conditions with unrestricted access to water and food. SKG mice aged 8–10 weeks were injected intraperitoneally with curdlan (Wako, #034‐09901) at a dose of 3 mg per mouse to induce disease. For the tail suspension experiment, SKG mice were randomly divided into two groups: the tail suspension (TS) group and the NTS group. In the TS group, the SKG mice were subjected to tail suspension (12 h/day) at the time of disease induction to alleviate mechanical stress on the hind limbs, whereas the SKG mice in the NTS group were kept in normal cages without any additional intervention after disease induction. For the CXCL5 neutralizing experiment, 3 µg of CXCL5 neutralizing antibody (R&D, #MAB433) was injected into the tendon entheses of SKG mice every 3 days, and the control group was injected with an equal volume of saline. Clinical scores for arthritis were recorded by two independent observers in a blinded manner as follows: 0 = normal; 0.1 = swelling or redness of one digit; 0.5 = mild swelling and/or redness of the wrist or ankle joints; 1 = moderate swelling and redness of the wrist or ankle joints; 1.5 = severe swelling of the entire joint; and 2 = ankylosis or deformity of the entire joint. After 4 weeks of induction and intervention, the mice were sacrificed, and the hind limbs and tendon entheses were dissected for subsequent experiments.

### 4.3 Tissue Dissociation and Single‐Cell RNA Sequencing

Achilles tendon entheses from SKG mice were dissected and transferred to sterile centrifuge tubes. The tissues were cut into 2 mm tissue blocks and digested with 0.5% collagenase type II (Thermo Fisher, #17101015) for 2 h in a 37°C constant temperature shaker. The cell suspension was filtered through a 40 µm cell strainer and then centrifuged at 400 × *g* for 6 min at room temperature, after which the supernatant was discarded to collect the dissociated cells. Red blood cell lysis buffer was added to resuspend the cells, which were then incubated for 5 min at room temperature. The cell suspension was centrifuged again at 400 × *g* for 6 min at room temperature, and the Dead Cell Removal Kit was used to remove dead cells. Cell viability was assessed with trypan blue, and samples with greater than 80% cell viability were subjected to single‐cell RNA sequencing using the 10× Genomics 3' Reagent Kit (v3.1). A single‐cell suspension was loaded onto a Chromium Controller and partitioned into gel bead‐in‐emulsion (GEM) units by a microfluidic cross‐system according to the manufacturer's protocol. Reverse transcription was performed to synthesize cDNA, followed by fragmentation, adapter ligation, PCR amplification and product purification. The libraries were sequenced on an Illumina instrument by NovelBio Technology.

### 4.4 Single‐Cell RNA Sequencing Analysis

CellRanger (v5.0.1) was used to process the raw data to generate a gene expression matrix. The Seurat package (v3.2.0) was used for further analysis, and cells with fewer than 200 detected genes, more than 6000 genes, or mitochondrial gene percentages exceeding 10% were filtered out. Multiple samples were integrated using the CCA algorithm to correct for batch effects. The gene expression dataset was normalized, and the top 2000 highly variable genes from the dataset were selected for PCA. A shared nearest neighbor graph was constructed using the first 10 principal components. Cells were clustered using the FindClusters function with a resolution parameter set to 0.5. Further dimensionality reduction was conducted via the tSNE algorithm. The marker genes of the clusters were identified via the FindAllMarkers algorithm, with thresholds set at logfc > 0.25, minimum expression proportion > 0.1 and adjusted *p* value < 0.05. Relevant clusters were extracted for further dimensionality reduction via uniform manifold approximation and projection (UMAP), and cell subtype marker genes were analyzed. Clusters identified as mixed cells were removed before downstream analysis. The clusterProfiler package (v3.11) was used to perform GO and Kyoto Encyclopedia of Genes and Genomes (KEGG) enrichment analyses, and terms with a *Q* value < 0.05 were considered significantly enriched. Pseudotime analysis was performed via Monocle2, and developmental trajectory inference was based on the DEGs of each cluster. Branched expression analysis modeling (BEAM) was used to identify genes with branch‐dependent expression, and significant genes with a *Q* value < 0.0001 were selected to generate a heatmap. Cell‒cell communication was assessed via the CellChat package (v1.5.0), and the CellChatDB database was used to infer the ligand‒receptor interactions between different cell clusters. A cell‒cell communication network was constructed by integrating the number of interactions and interaction strength of each cluster. Two‐dimensional visualization of the dominant senders and receivers was achieved on the basis of the total outgoing and incoming communication probabilities associated with each cell cluster. Differential numbers of interactions and differential interaction strengths between the tail suspension group and the NTS group in the communication network were visualized as heatmaps. Significant ligand‒receptor interactions with *p* values < 0.05 are presented in the scatter plot.

### 4.5 Micro‐CT Scanning

The harvested samples were fixed with 4% paraformaldehyde and scanned via a Siemens Inveon CT scanner. The segmented data were reconstructed as 3D CT images via RadiAnt DICOM Viewer software (v5.0.2).

### 4.6 Histological Staining

The harvested samples were fixed with 4% paraformaldehyde for 24 h and decalcified in 0.5 M EDTA solution for 2 weeks. The samples were subsequently dehydrated, paraffin‐embedded and sectioned. The sections were deparaffinized, rehydrated and stained with hematoxylin (Servicebio, #G1004) for 10 min, followed by immersion in 1% alcoholic hydrochloric acid solution for 3 s. After washing with PBS, the sections were stained with eosin (Servicebio, #G1002) for 5 min, dehydrated and sealed. Images were captured with a microscope.

### 4.7 Multiplex Immunofluorescence Staining

The sections were deparaffinized and rehydrated, and then heat‐induced antigen retrieval was performed using Tris‐EDTA buffer (pH 9.0) at 65°C overnight. After being washed with PBS, the sections were incubated in 3% hydrogen peroxide solution for 20 min at room temperature to inactivate endogenous peroxidases and subsequently incubated with 5% normal goat serum for 1 h at room temperature to block nonspecific binding. The sections were subsequently incubated with primary antibodies at 4°C overnight. On the second day, the sections were incubated with HRP‐conjugated secondary antibodies for 1 h at room temperature, followed by incubation with fluorescein‐labeled tyramide for 20 min. Then, the sections were treated with antibody elution buffer (Panovue, #1010800) for 10 min to elute the conjugated antibodies. After being washed with PBS, the sections were subjected to the next round of staining. Nuclei were stained with DAPI (Servicebio, #G1012), and images were captured with a digital pathology slide scanner (KFBIO). The immunofluorescence‐positive cells were quantified by two independent investigators, who were blinded to the experimental information. The spatial distance between fibroblasts and neutrophils was calculated by the HALO proximity analysis algorithm. The following antibodies and fluoresceins were used: anti‐mouse Pdgfra (Invitrogen, #14‐1401‐82), anti‐human PDGFRa (Abcam, #ab20349), anti‐mouse/human Sdc1 (Proteintech, #10593‐1‐AP), anti‐mouse/human SOX5 (Abcam, #ab94396), anti‐mouse Ly6g (BD, #551459), anti‐mouse/human Cxcr4 (Abcam, #ab124824), anti‐mouse/human H3cit (Abcam, # ab281584), anti‐mouse/human MPO (R&D, #AF3667), donkey anti‐goat IgG Alexa Fluor 647 (Abcam, #ab150131), HRP‐conjugated goat anti‐rabbit IgG (Servicebio, #GB23303), HRP‐conjugated goat anti‐rat IgG (Servicebio, #GB23302), iF488‐tyramide (Servicebio, #G1231), iF555‐tyramide (Servicebio, #G1233), iF594‐tyramide (Servicebio, #G1242) and iF647‐tyramide (Servicebio, #G1232).

### 4.8 Flow Cytometry

Tissue dissociation and digestion were performed as described above. The cells were incubated with reagents from the Zombie Violet Fixable Viability Kit (BioLegend, #423113) for 15 min at room temperature and washed with PBS. The cells were subsequently incubated with specific antibodies for 30 min at 4°C in the dark and then washed with PBS. The samples were evaluated with a BD FACSCelesta cytometer. The following antibodies were used: anti‐mouse Pdgfra‐APC (BioLegend, #135907), anti‐mouse Sdc1‐PE (BioLegend, #142503), anti‐human SDC1‐APC (BioLegend, #352307), anti‐mouse Ly6g‐APC (BioLegend, #127613), anti‐mouse CXCR4‐PE (BioLegend, #146505), anti‐human CXCR4‐PE (BioLegend, #306505), anti‐mouse CD62L‐APC (BioLegend, #161218), and anti‐human CD62L‐APC (BioLegend, #304810).

For CXCR4^hi^ neutrophil sorting, mouse tendon entheses were dissociated and digested as described above. The cells were incubated with reagents from the Zombie Violet Fixable Viability Kit (BioLegend, #423113) for 15 min at room temperature and washed with PBS. Then, the cells were incubated with anti‐mouse Ly6g‐APC (BioLegend, #127613) and anti‐mouse CXCR4‐PE (BioLegend, #146505) antibodies for 30 min at 4°C in the dark and washed with PBS. For SDC1^+^ fibroblast sorting, human entheseal fibroblasts were isolated and cultured as described above. These cultured cells were digested and incubated with anti‐human SDC1‐APC (BioLegend, #352307) and anti‐human Pdgfra‐APC (BioLegend, #323511) antibodies for 30 min at 4°C in the dark and washed with PBS. CXCR4^hi^ LY6G‐positive neutrophils or SDC1‐positive fibroblasts were sorted with a flow cytometry cell sorter (BD FACSAria III). The gating strategies used in the flow cytometry were shown in Figure S8.

### 4.9 ELISA

Tendon entheses from SKG mice were isolated to assess CXCL5 with a CXCL5 ELISA Kit (R&D Systems, #MX000) according to the manufacturer's instructions. Briefly, tendon entheses were homogenized in PBS, lysed in lysis buffer at room temperature for 30 min, and then centrifuged to remove debris. The lysates and standard were added to a microplate and incubated for 2 h at room temperature. The samples were aspirated and washed five times with washing buffer, and a mouse CXCL5 conjugate was added to each well and incubated for 2 h at room temperature. After five washes, the substrate mixture was added to the wells and incubated for 30 min at room temperature. The stop solution was subsequently added to the wells to stop the reaction. The optical density was measured with a microplate reader set to 450 nm, and a standard curve was created using standard samples. Accurate CXCL5 concentrations were calculated based on the standard curve, and data was normalized by per mg tissue.

### 4.10 Isolation and Culture of Entheseal Fibroblasts

Spinal entheseal tissues were obtained during spine surgeries on AS patients and trauma patients to isolate human entheseal fibroblasts. Mouse tendon entheses were dissociated to isolate mouse entheseal fibroblasts. The harvested tissues were transferred to sterile centrifuge tubes, cut into 2 mm blocks, and then digested with 0.1% collagenase type II (Thermo Fisher, #17101015) for 12 h at 37°C. The tissue suspension was collected and centrifuged at 400 × *g* for 10 min at room temperature, and the supernatant was discarded to collect the digested tissues. After being washed with sterile PBS, the digested tissues were resuspended in high‐glucose DMEM (GIBCO, #C11995500BT) containing 10% fetal bovine serum (FBS, TIANHANG, #13011‐8611) and 1% penicillin‒streptomycin and then placed in cell culture flasks at 37°C in a 5% CO_2_ atmosphere for 3 days for the tissue blocks to adhere to the bottom of the flasks. The nonadherent tissues were removed, and the culture medium was replaced every 3 days. When the primary fibroblasts reached 80%–90% confluence, the cells were digested with 0.25% trypsin with 0.53 mM EDTA and reseeded in new flasks for experiments.

### 4.11 Isolation of Human Neutrophils

Neutrophils were isolated from the peripheral blood of healthy adult donors through density gradient centrifugation, and erythrocytes were lysed with red blood cell lysis buffer (Solarbio, #R1010). After three washes with PBS, the purified neutrophils were resuspended in RPMI 1640 culture medium supplemented with 10% FBS.

### 4.12 Isolation of Mouse Bone Marrow Neutrophils

Wild‐type BALB/c mice (8–10 weeks old) were euthanized by cervical dislocation under anesthesia. Femurs were surgically excised, and bone marrow cells were flushed with ice‐cold PBS. The cell suspension was centrifuged, and erythrocytes were lysed with red blood cell lysis buffer (Solarbio, #R1010). Neutrophils were then isolated via a mouse neutrophil isolation kit (BEAVER, #70907) following the manufacturer's protocol.

### 4.13 Neutrophil Migration Assay

Neutrophil chemotaxis was evaluated using 24‐well Transwell chambers with 5‐µm pore polycarbonate membranes (Corning, #3421). For fibroblast‐induced migration, primary human or mouse entheseal fibroblasts (2 × 10^4^) were seeded in the lower chambers with or without 0.5 µg/mL anti‐CXCL5 neutralizing antibody (R&D, #AF254, #MAB433). For cytokine‐driven migration, 10 nM recombinant CXCL5 (PeproTech, #300‐22, #250‐17) was loaded into the lower chambers. Purified neutrophils of the corresponding species (2 × 10^5^) were added to the upper chambers. Both sets of cells were cultured in RPMI 1640 culture medium without FBS. After 3 h of incubation, the migrated neutrophils in the lower chamber were collected and counted by flow cytometry.

### 4.14 Coculture of Neutrophils and Entheseal Fibroblasts

Neutrophils were cocultured with primary entheseal fibroblasts in 24‐well Transwell chambers with 0.4‐µm pore polycarbonate membranes (Corning, #3413). Primary human or mouse entheseal fibroblasts (2 × 10^4^) were seeded in the upper chambers 24 h prior to coculture, and purified neutrophils of the corresponding species (2 × 10^5^) were seeded in the lower chambers with or without 0.5 µg/mL anti‐CXCL5 neutralizing antibody. Following 6 h of coculture, neutrophils were collected to assess the CXCR4^high^ neutrophil ratio and CD62L shedding by flow cytometry or to evaluate intracellular ROS production and NETosis as described below.

### 4.15 ROS Detection

Intracellular ROS production was quantified with an ROS assay kit (Beyotime, #S0033S) following the manufacturer's instructions. Briefly, neutrophils were loaded with a 10 µM DCFH‐DA fluorescent probe at 37°C for 20 min and subsequently stimulated with 2 nM PMA (MultiSciences, #70‐CS0001) at 37°C for 2 h. Then, intracellular ROS were immediately detected by flow cytometry.

### 4.16 NETosis Assay

To visualize NETs in vitro, neutrophils were stimulated with 2.5 nM PMA for 4 h to induce NETosis. Neutrophils were subsequently fixed with 4% paraformaldehyde and permeabilized with 0.2% Triton X‐100. The cells were subsequently blocked with 2% BSA for 1 h at room temperature and incubated with anti‐mouse/human H3cit (Abcam, #ab281584) and anti‐mouse/human MPO (R&D, #AF3667) antibodies at 4°C overnight. The samples were incubated with the secondary antibodies donkey anti‐goat IgG Alexa Fluor 647 (Abcam, #ab150131) and donkey anti‐rabbit IgG Alexa Fluor 488 (Abcam, #ab150073). DAPI was used to stain the nuclei and the extracellular DNA scaffolds. Images were obtained by confocal microscopy (LSM980 Zeiss).

### 4.17 Quantification of MPO‐DNA

Circulating MPO‐DNA in SKG mouse plasma was quantified using a modified capture ELISA protocol. SKG mice were euthanized by cervical dislocation under anesthesia, and whole blood samples were collected and centrifuged at 1000 × *g* for 10 min at 4°C to isolate the plasma. A total of 5 µg/mL anti‐MPO antibody (R&D, # AF3667) was added to the 96‐well plates overnight at 4°C as the capture antibody. After blocking with 1% BSA, plasma combined with peroxidase‐labeled anti‐DNA monoclonal antibody (Roche, #11774425001) was added, and the mixture was incubated at room temperature for 2 h. Subsequently, peroxidase substrate was added, and the mixture was incubated at 37°C for 40 min. The optical density was detected at 405 nm via a Thermo Varioskan lux instrument.

### 4.18 RNA Sequencing and Data Analysis

Total RNA was extracted from human spinal entheseal fibroblasts by TRIzol (Invitrogen, #15596‐026) according to the manufacturer's instructions. RNA integrity was evaluated with the Agilent Bioanalyzer 2100 system. A cDNA library was constructed, and paired‐end 150‐base reads were generated by the Beijing Genomics Institute by the BGISEQ‐500 platform. Reads containing adaptor contamination or low‐quality reads and those with an unknown base ratio of more than 5% were filtered out with SOAPnuke (v1.5.6) to generate clean reads. The clean reads were then mapped to a reference genome (GRCh38.p12 for humans or GRCm38.p6 for mice) via HISAT2 (v2.0.4). RSEM (v1.3.1) was used to calculate the expression levels of genes, and DEGs were analyzed by DESeq2 (v1.4.5) with thresholds of |log2Fold Change| > 1 and a *Q* value < 0.05. The clusterProfiler package (v3.11) was used to perform GO and KEGG analyses as described above. Heatmaps and volcano plots were generated with OmicStudio tools. Gene set variation analysis was conducted via the GSVA package (v1.46.0), with the method set to ZSCORE and kcdf set to Gaussian.

### 4.19 Mechanical Strain Stimulation In Vitro

Human spinal entheseal fibroblasts or mouse tendon entheseal fibroblasts (1 × 10^5^ cells per well) were seeded on BioFlex culture plates (Flexcell, #BF‐3001C) and cultured at 37°C in a 5% CO_2_ atmosphere for 24 h. Then, mechanical strain stimulation was applied with the FX5000 Tension System (Flexcell), and the mechanical strain parameters were set to 5% elongation, 0.5 Hz and a sinusoidal waveform, with a stimulation duration of 6 h per day for a total of 2 days. For the control samples, cells were seeded on BioFlex culture plates and cultured under the same conditions without any mechanical strain stimulation.

### 4.20 RNA Interference

Small interfering RNAs (siRNAs) targeting human or mouse SOX5 and a negative control were purchased from GenePharma. After the fibroblasts reached 70–80% confluence, they were transfected with siRNAs using OptiMEM (GIBCO, #31985070) and Lipofectamine RNAiMAX (Invitrogen, #13778150) according to the manufacturer's instructions. The knockdown efficiency was analyzed via qRT‒PCR and Western blotting after 72 h. The sequences of the siRNAs utilized in this study are listed in Table S1.

### 4.21 CUT&Tag Assay

The CUT&Tag assay was performed with a NovoNGs CUT&Tag 4.0 High‐Sensitivity Kit (Novoprotein, #N259‐YH01‐01A) following the manufacturer's instructions. Briefly, a total of 1 × 10^5^ cells were harvested and bound to ConA magnetic beads. The cells were incubated with a primary H3K27ac antibody (Abcam, #ab177178) or a primary H3K4me1 antibody (Abcam, #ab176877) at 4°C overnight and then incubated with goat anti‐rabbit IgG H&L (Novoprotein, #N269) at room temperature for 1 h. After washing with wash buffer, the cells were incubated with pAG‐Transposome at room temperature for 1 h. Subsequently, the cells were washed with wash buffer and incubated with tagmentation buffer at 37°C for 1 h, after which the reaction was terminated with stop buffer at 55°C for 10 min. DNA was extracted with tagmented DNA extraction beads, and PCR was performed with index primers to construct the CUT&Tag library. The PCR products were purified with DNA purification beads. Library quality was evaluated by the Agilent Bioanalyzer 2100 system.

### 4.22 CUT&Tag Sequencing and Analysis

The CUT&Tag libraries were sequenced on the Illumina NovaSeq 6000 platform by Novogene Technology, and paired‐end 150‐base reads were generated. The sequencing adaptors and low‐quality reads were removed via TrimGalore (v0.6.6) with the parameters ‐q 20 –phred33–stringency 3. The clean reads were then mapped to a reference genome (GRCh38.p12). MACS2 (v.2.1.1) was used to call peaks with the parameter ‐q 0.05 –call‐summits –nomodel–shift ‐100 –extsize 200 –keep‐dup all. Heatmaps of H3K27ac and H3K4me1 signals were generated with the computeMatrix and plotHeatmap commands in deepTools (v2.3.6.0).

### 4.23 CUT&Tag‐qPCR Assay

An anti‐H3K27ac antibody (Abcam, #ab177178), anti‐SOX5 antibody (Abcam, #ab 94396), and rabbit IgG (Abcam, #ab172730) were used as primary antibodies for the CUT&Tag‐qPCR assay. CUT&Tag libraries were constructed as described above and then used as templates to perform qPCR in triplicate. The fold enrichment of H3K27ac signals at the indicated marker genes was calculated via the 2^−ΔΔCt^ method, with the data normalized to the values of the IgG controls. The primers used for CUT&Tag‒qPCR were listed in Table S2.

### 4.24 Quantitative Analysis of H3K27ac and H3K4me1 Levels

The total H3K27ac and H3K4me1 levels of fibroblasts were detected using EpiQuik Global Acetyl Histone H3K27 Quantification Kit and EpiQuik Global Pan‐Methyl Histone H3K4 Quantification Kit. Briefly, cells were washed with PBS, and the total histone was extracted from the cells using Histone Extraction Kit (Abcam, #ab113476). A total of 100 ng histone was subjected to ELISA assay for global acetyl histone H3K27 and global methyl histone H3K4 according to the manufacturer's instruction.

### 4.25 Construction and Transfection of rAAV9.HAP‐1

rAAV9.HAP‐1 was designed as previously described and synthesized by OBiO Technology. A specific sequence (ATGAGCTTCCACCAGTTCGCCCGCGCCACCCTGGCCAGC) encoding the transduction peptide HAP‐1 was inserted at the N‐terminus of the AAV9‐VP2 open reading frame (ORF). This modified packaging plasmid was used to construct rAAV9.HAP‐1. Five‐week‐old SKG mice were used for the in vivo intervention experiment, and rAAV9.HAP‐1 was labeled with mScarlet. Subsequently, rAAV9.HAP‐1‐sh‐SOX5 (AV‐SOX5) or rAAV9.HAP‐NC (AV‐NC) was locally injected around the tendon entheses, with a virus titer of 1 × 10^13^ VG/ml and an injection volume of 10 µl per site. Three weeks later, the SKG mice were subjected to intraperitoneal injection of curdlan (3 mg/mouse) to induce disease, and the clinical arthritis scores were determined as described above. After four weeks of induction, the mice were sacrificed, and specimens were harvested for subsequent experiments.

### 4.26 RNA Extraction, Reverse Transcription, and Quantitative Real‐time PCR

Total RNA was extracted from fibroblasts with TRIzol (Invitrogen, #15596‐026) according to the manufacturer's instructions and reverse transcribed into cDNA with Evo M‐MLV RT Master Mix (AG, #11706). Quantitative real‐time PCR was conducted with the SYBR Green Premix Pro Taq HS qPCR Kit (AG, #11718) on the Applied Biosystems 7500 Real‐Time PCR System. The 2^−ΔΔCt^ method was used to calculate the relative expression levels of target genes, and GAPDH was used as the reference gene to normalize the data. Each qRT‒PCR was performed in triplicate. The qRT‒PCR primers used are listed in Table S2.

### 4.27 Protein Extraction and Western Blotting

The cells were lysed in RIPA buffer (CWBIO, #CW2333S) containing protease inhibitors (CWBIO, #CW2200S) at 4°C for 30 min. The lysates were subsequently centrifuged at 12,000 × *g* for 30 min at 4°C to collect the supernatant, and a Pierce BCA protein assay kit (CWBIO, #CW0014S) was used to quantify the protein concentration. The harvested protein was subsequently mixed with loading buffer (Beyotime, #P0015) and boiled to denature the protein. Equal amounts of protein were loaded and separated via sodium dodecyl sulfate–polyacrylamide gel electrophoresis (SDS–PAGE) and then transferred to PVDF membranes (Millipore, #IPVH00010). The PVDF membranes were blocked with 5% nonfat milk and incubated with primary antibodies at 4°C overnight, followed by incubation with HRP‐conjugated secondary antibodies for 1 h at room temperature. Immunolabeling was detected by the use of a chemiluminescent HRP substrate (Millipore, #WBKLS0500), and protein levels were quantified with ImageJ software. The following antibodies were used in this study: anti‐SOX5 (Abcam, #ab94396) and anti‐GAPDH (Cell Signaling Technology, #5174S).

### 4.28 Dual‐Luciferase Reporter Assay

The enhancer sequences of *Sdc1* or *Cxcl5* were synthesized and cloned into a pGL3‐promoter vector. 293 T cells were separately transfected with above plasmids followed by transfection with siRNAs to knockdown SOX5 using Lipofectamine RNAiMAX (Invitrogen). pRL‐TK plasmids were transfected as an internal control. Luciferase activities were measured using the Dual‐Luciferase Reporter Assay System (Promega, E1910, Massachusetts, USA). Firefly luciferase activity was normalized to Renilla luciferase activity for each sample.

### 4.29 Statistical Analysis

Statistical analysis was performed with GraphPad Prism 8.0 software. Two‐tailed Student's *t* tests were used to compare two experimental groups. One‐way ANOVA with Bonferroni's multiple comparison test was used for comparisons among three or more groups. The correlation analysis was assessed using Spearman's rank correlation coefficient. The data are presented as the means ± standard deviations (SDs), and a *p* value < 0.05 was considered to indicate significance. Schematic diagrams were created with BioRender.com.

## Funding

This study was financially supported by National Natural Science Foundation of China (82272448, 82402764), China Postdoctoral Science Foundation (2024M763796), Guangdong Natural Science Foundation (2023A1515030026, 2023A1515111078), Shenzhen Science and Technology Program (JCYJ20240813150720026, RCBS20221008093103013), Shenzhen Medical Research Fund (A2403047) and Futian Healthcare Research Project (FTWS2022016, FTWS016).

## Ethics statement

This study was approved by the Ethics Committee of the Eighth Affiliated Hospital, Sun Yat‐Sen University, Guangzhou, China (ZDBY‐IIT‐202403‐47). The experiments involving mice were approved by the Institutional Animal Care and Use Committee of Sun Yat‐Sen University, Guangzhou, China (SYSU‐IACUC‐2023‐001241).

## Conflicts of Interest

The authors declare no conflicts of interest.

## Supporting information




**Supporting File 1**: advs74230‐sup‐0001‐SuppMat.docx.

**Supporting File 2**: advs74230‐sup‐0002‐DataFile.xlsx.

## Data Availability

The data of RNA sequencing generating in this study has been deposited in the NCBI BioProject database under accession code PRJNA1129055, PRJNA1131764, PRJNA1131766. All other data supporting the findings of this study are available within the article and its supplementary data.
