# Supplementary material for: Mechanical Strain‐Programmed SDC1+ Sheath Fibroblasts Trigger CXCR4hi Neutrophil‐Mediated Enthesitis in Ankylosing Spondylitis
Source: Adv Sci (Weinh). 2026 Feb 4;13(21):e20617. doi: 10.1002/advs.202520617 (PMC13073239; doi:10.1002/advs.202520617)
Supplement: Supplementary file 1 — Supporting File 1: advs74230‐sup‐0001‐SuppMat.docx. [file ADVS-13-e20617-s002.docx]

**Supplementary material for manuscript**

**Mechanical Strain-Programmed SDC1^+^ Sheath Fibroblasts Trigger CXCR4^hi^ Neutrophil-Mediated Enthesitis in Ankylosing Spondylitis**

Jiajie Lin^1#^, Zepeng Su^1#^, Yipeng Zeng^1#^, Yi Zhou^1^, Chenying Zeng^2^, Weihao Zhang^1^, Qibo Li^1^, Zipeng Xiao^1^, Zibin Chen^1^, Ziqian Liu^1^, Yangfeng Lin^1^, Guan Zheng^1,3*^, Wenhui Yu^1,3*^, Zhongyu Xie^1,2,3*^

**
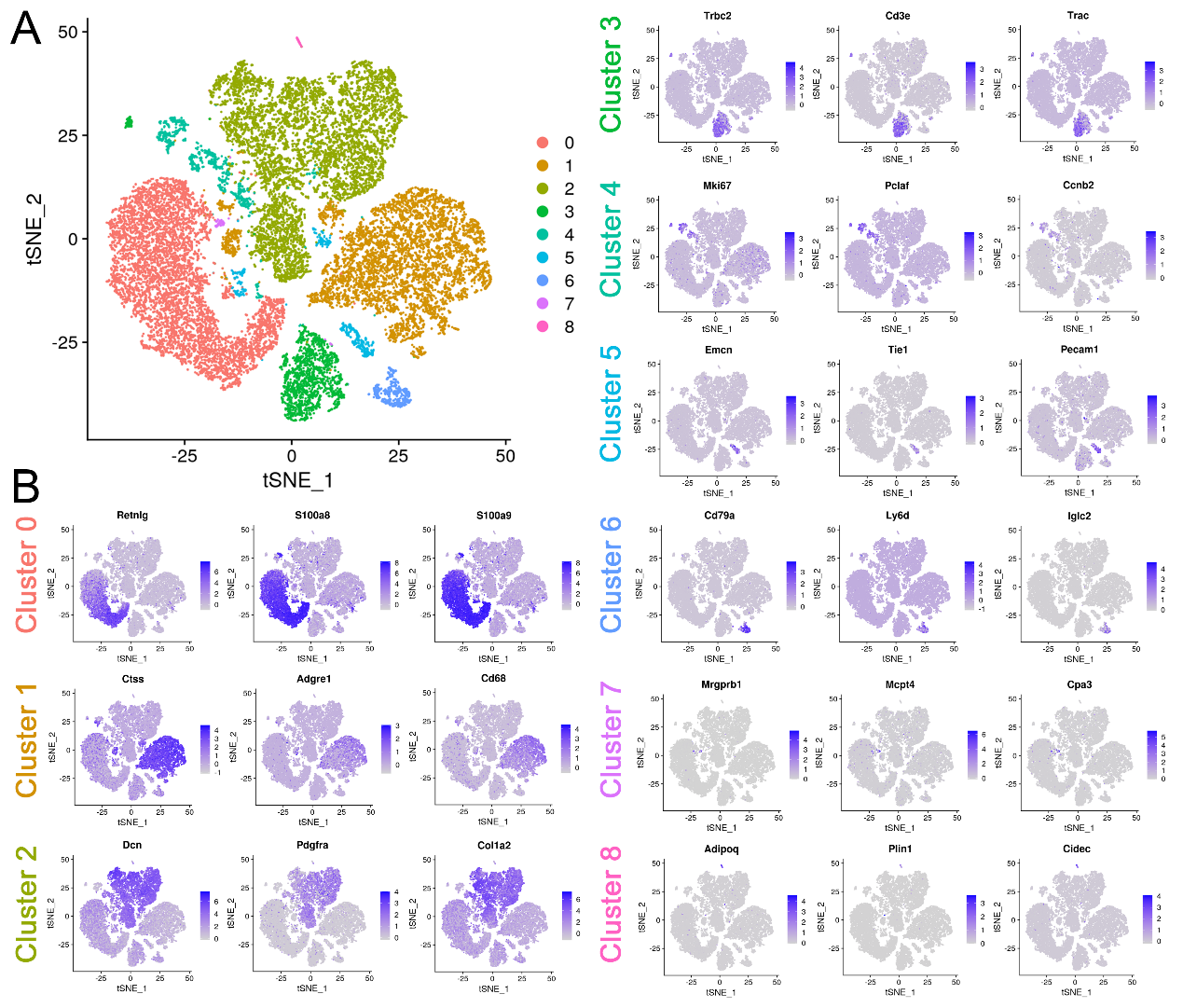
**

**Supplementary Figure 1. Single-cell landscape and cluster marker genes of entheses from SKG mice in the NTS and TS groups**

(A) t-SNE view of 23628 cells color-coded according to nine cell types. (B) t-SNE visualization color-coded according to the expression (gray to purple) of marker genes of each subcluster.

**
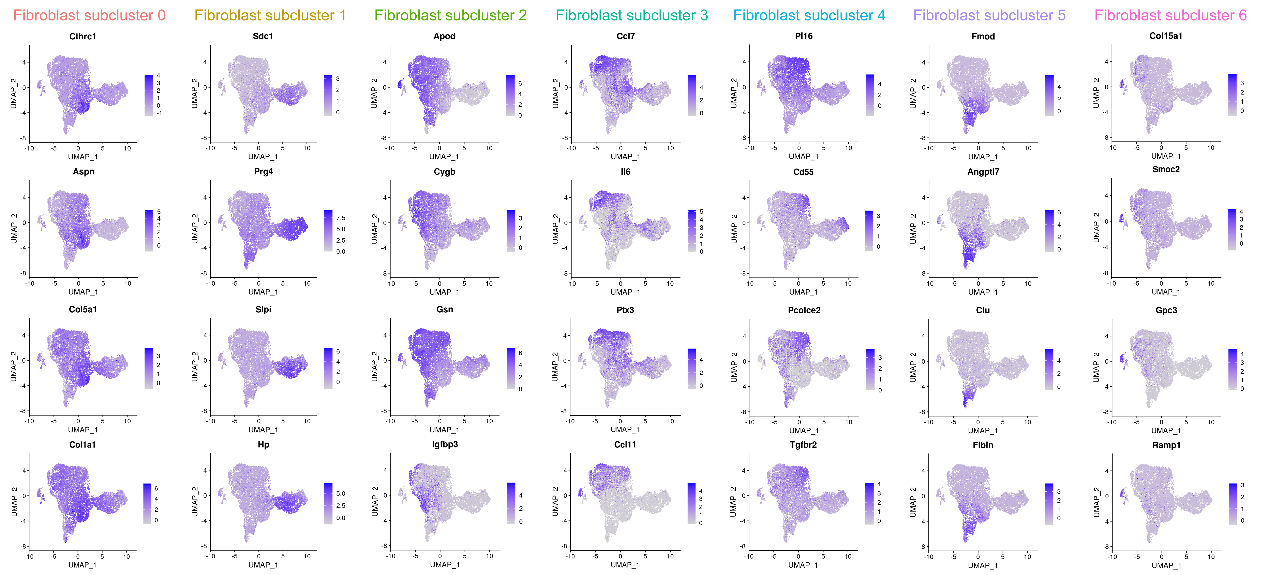
**

**Supplementary Figure 2. The marker genes of the fibroblast subcluster**

UMAP visualization color-coded according to the expression (gray to purple) of marker genes of each fibroblast subcluster.

**
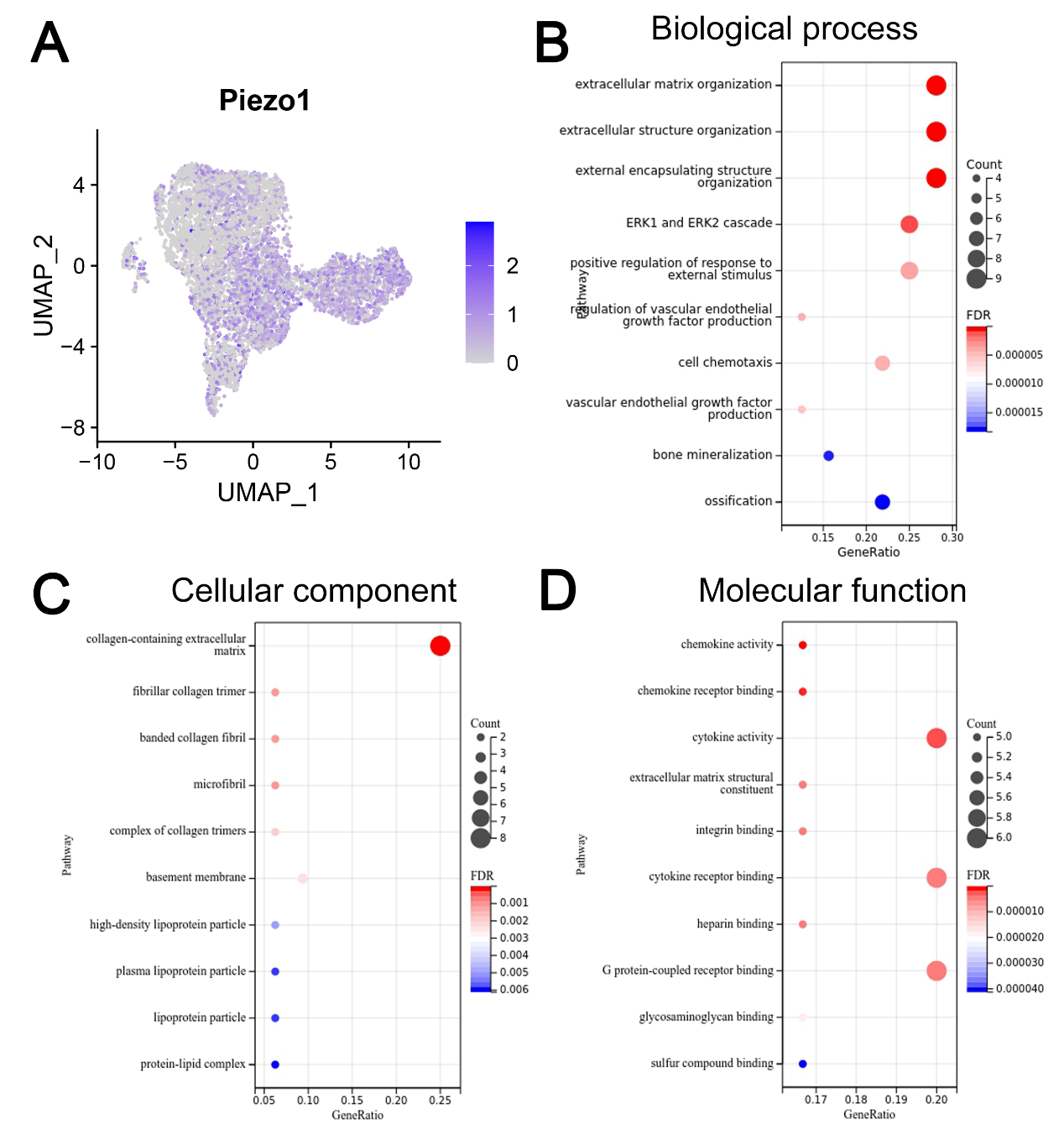
**

**Supplementary Figure 3. Characteristics and functional enrichment analysis of the differentially expressed genes of SDC1^+^ sheath fibroblasts**

(A) UMAP visualization color-coded according to the expression (gray to purple) of *Piezol1* in the fibroblast cluster. (B) GO analysis of the biological process terms enriched in the DEGs of SDC1^+^ sheath fibroblasts. (C) Cellular component terms enriched in the DEGs of SDC1^+^ sheath fibroblasts according to GO analysis. (D) Molecular function terms enriched in the DEGs of SDC1^+^ sheath fibroblasts according to GO analysis.

**
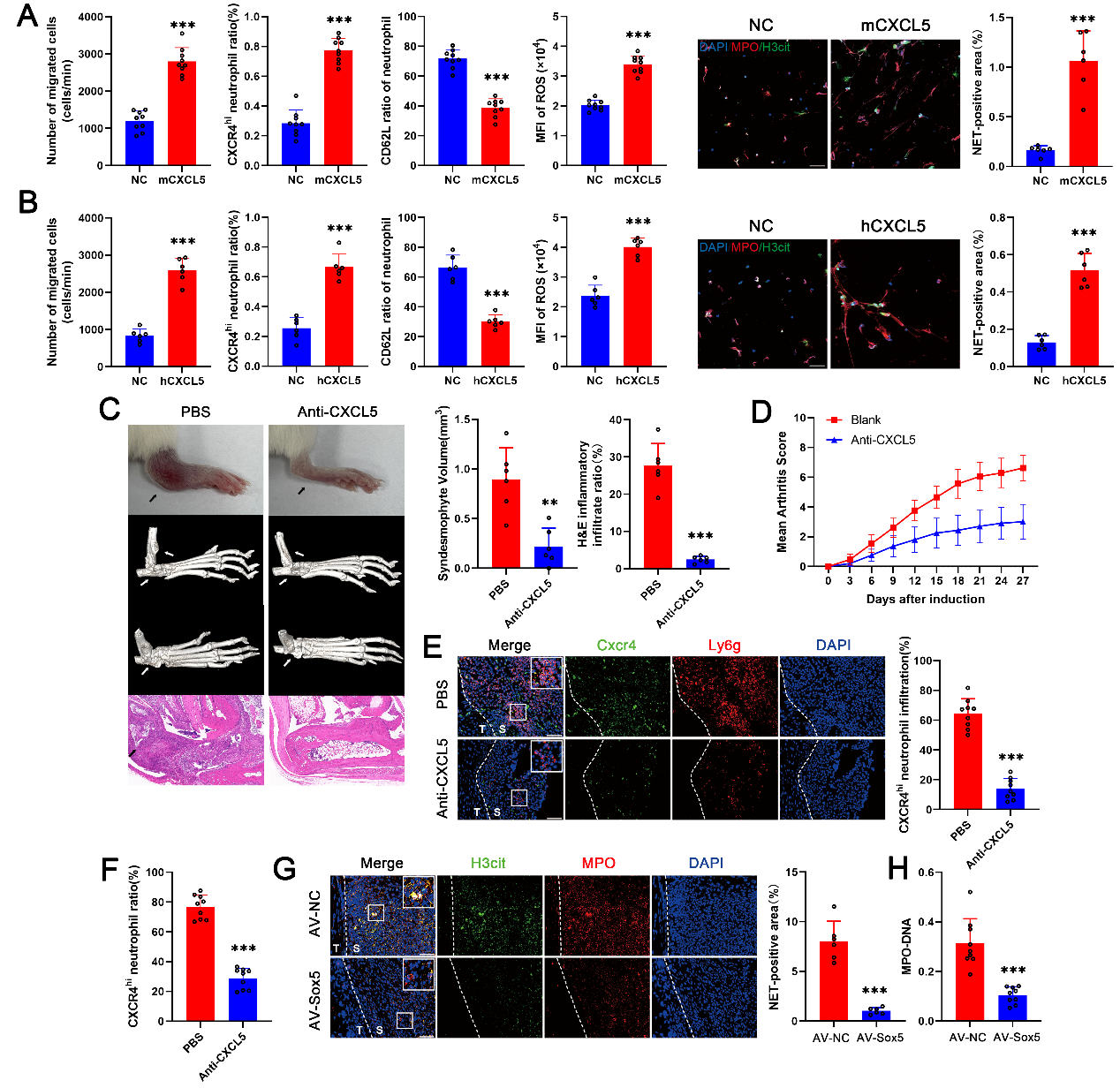
**

**Supplementary Figure 4. The critical role of CXCL5 in regulating neutrophils functions and in the treatment of AS model**

(A) Exogenous mouse CXCL5 promoted the mice neutrophils migration, increased the CXCR4^hi^ ratio, ROS levels and NETs formation (positive for MPO and H3cit) and decreased the CD62L ratio (n=9). Scale bar, 50 μm. (B) Exogenous human CXCL5 promoted the human neutrophils migration, increased the CXCR4^hi^ ratio, ROS levels and NETs formation (positive for MPO and H3cit) and decreased the CD62L ratio (n=6). Scale bar, 50 μm. (C) The degree of swelling, number of syndesmophytes and severity of inflammatory infiltration in the entheses were alleviated after CXCL5 neutralizing antibody treatment (Black and white arrows). Scale bar, 200 μm. (D) The mean arthritis score was lower in the anti-CXCL5 group (n=9). (E) Immunofluorescence image showing the reduced ratio of CXCR4^hi^ neutrophils in the entheses of CXCL5 neutralizing antibody-treated SKG mice (n=9). (F) Flow cytometry showing the decreased ratio of CXCR4^hi^ neutrophils in CXCL5 neutralizing antibody-treated SKG mice (n=9). (G) Immunofluorescence image showing the decreased NETs (positive for H3cit and MPO) levels in the entheses of CXCL5 neutralizing antibody-treated SKG mice. (H) The MPO-DNA levels in the peripheral blood were lower in the anti-CXCL5 group (n=9. The values are presented as the means ± SDs. ** indicates P<0.01 and *** indicates P<0.001. S indicates the tendon sheath and T indicates the tendon. Scale bar of immunofluorescence, 50 μm.

**
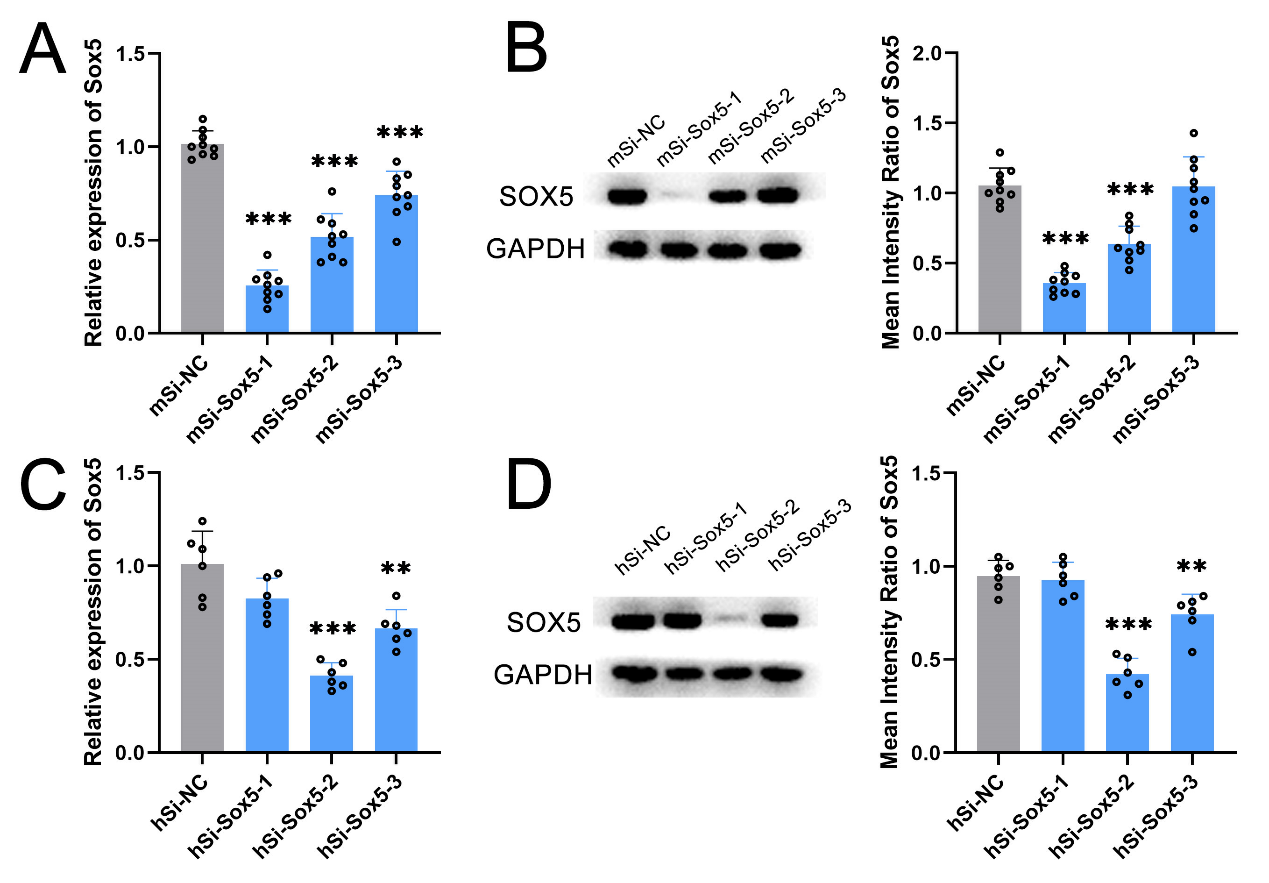
**

**Supplementary Figure 5. Interference efficiency of *Sox5* siRNA in human and mouse fibroblasts**

(A) qRT‒PCR analysis showing the inhibitory efficiency of mSi-Sox5 at the gene level (n=9). (B) Western blot showing the inhibitory efficiency of mSi-Sox5 at the protein level (n=9). (C) qRT‒PCR analysis showing the inhibitory efficiency of hSi-Sox5 at the gene level (n=6). (D) Western blot showing the inhibitory efficiency of hSi-Sox5 at the protein level (n=6). The values are presented as the means ± SDs. ** indicates P<0.01 and *** indicates P<0.001.

**
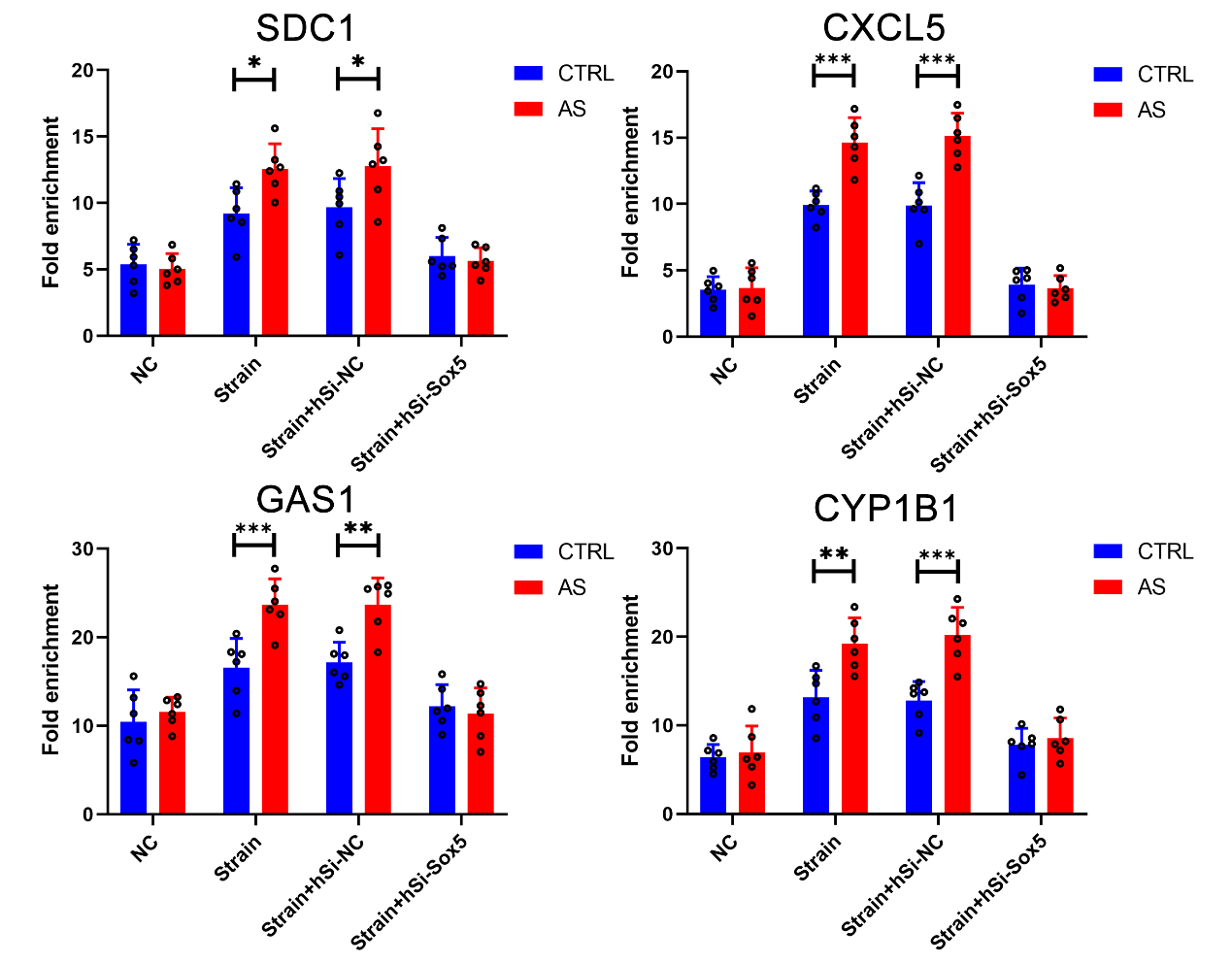
**

**Supplementary Figure 6. H3K27ac modification levels of SDC1^+^ sheath fibroblast marker genes in fibroblasts from AS patients.**

CUT&Tag-qPCR revealed increased H3K27ac abundance levels of *SDC1*, *CXCL5*, *GAS1* and *CYP1B1* in fibroblasts isolated from the spine entheses of AS patients under strain conditions, and these changes were reversed by transfection of hSi-Sox5. The values are presented as the means ± SDs. * indicates P<0.05, ** indicates P<0.01, *** indicates P<0.001.


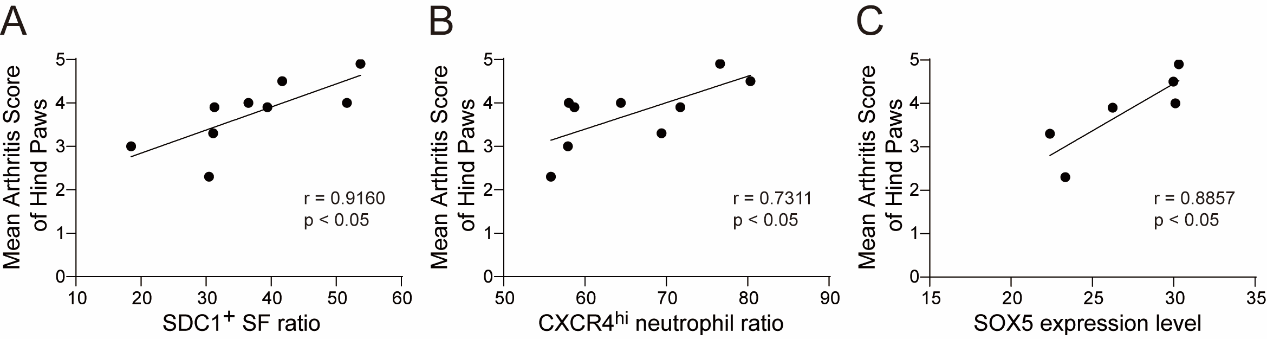


**Supplementary Figure 7. Correlation analysis of the SDC1⁺ sheath fibroblasts ratio/CXCR4^hi^ neutrophils ratio/SOX5 expression levels and the arthritis score of SKG mice.**

(A) The SDC1⁺ sheath fibroblasts ratio were all positively correlated to the mean arthritis score of diseased SKG mice (n=9). (B) The CXCR4^hi^ neutrophils ratio were all positively correlated to the mean arthritis score of diseased SKG mice (n=9). (C) The SOX5 expression levels of fibroblasts were all positively correlated to the mean arthritis score of diseased SKG mice (n=6).


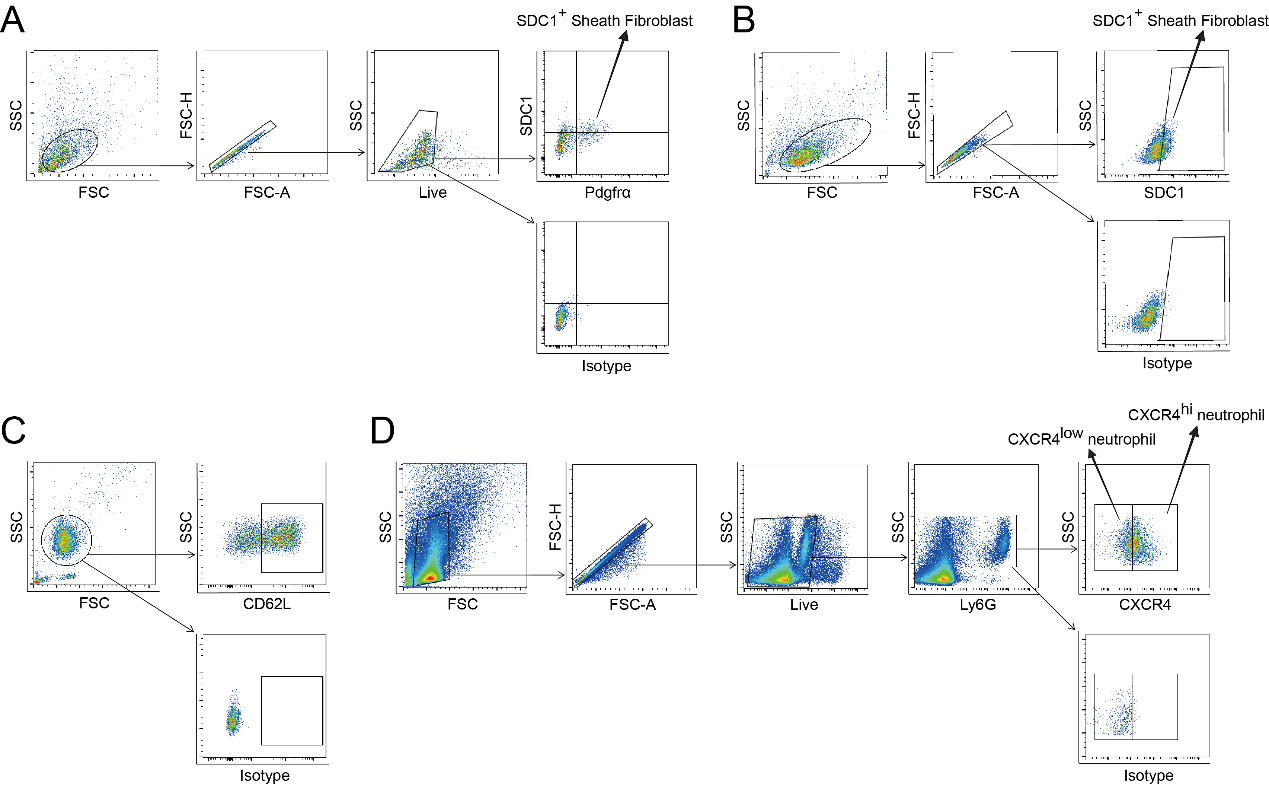


**Supplementary Figure 8. Gating strategies used in the flow cytometry.**

(A) Gating strategy for identification of SDC1+ sheath fibroblasts in the entheses of mice. (B) Gating strategy for identification of SDC1+ fibroblasts in cultured human entheseal fibroblasts. (C) Gating strategy for determination of the activation of human neutrophils. (D) Gating strategy for identification of CXCR4hi neutrophils and CXCR4low neutrophils in the entheses of mice.

**Table S1 siRNA sequences**

| Targets | |  | Sequence (5’ to 3’) |
| --- | --- | --- | --- |
| Mouse SOX5 siRNA-1 | | sense | GCUCAUGGGUAUGAUCAAUTT |
|  |  | antisense | AUUGAUCAUACCCAUGAGCTT |
| Mouse SOX5 siRNA-2 | | sense | GCUGCCGCCAUUGAUGAUUTT |
|  |  | antisense | AAUCAUCAAUGGCGGCAGCTT |
| Mouse SOX5 siRNA-3 | | sense | CCGUGUCUCCUACCAGCAUTT |
|  | | antisense | AUGCUGGUAGGAGACACGGTT |
| Human SOX5 siRNA-1 | | sense | GCCAUAUUAUGAGGAGCAATT |
|  |  | antisense | UUGCUCCUCAUAAUAUGGCTT |
| Human SOX5 siRNA-2 | | sense | GCUGCCGCCAUUAAUGAUUTT |
|  |  | antisense | AAUCAUUAAUGGCGGCAGCTT |
| Human SOX5 siRNA-3 | | sense | CCUGUUAUCCAGAGCACUUTT |
|  |  | antisense | AAGUGCUCUGGAUAACAGGTT |

**Table S2 Primers for qPT-PCR and CUT&Tag-qPCR**

| Primers | Sequence (5’ to 3’) | Application |
| --- | --- | --- |
| Mouse SOX5-forward | CTGCCGCCATTGATGATTCC | qRT-PCR |
| Mouse SOX5-reverse | CCAGCCTTGTAGCTGAAACCA | qRT-PCR |
| Mouse GAPDH-forward | AGGTCGGTGTGAACGGATTTG | qRT-PCR |
| Mouse GAPDH-reverse | TGTAGACCATGTAGTTGAGGTCA | qRT-PCR |
| Human SDC1-forward | ACGGCTATTCCCACGTCTC | qRT-PCR |
| Human SDC1-reverse | TCTGGCAGGACTACAGCCTC | qRT-PCR |
| Human CXCL5-forward | AGCTGCGTTGCGTTTGTTTAC | qRT-PCR |
| Human CXCL5-reverse | TGGCGAACACTTGCAGATTAC | qRT-PCR |
| Human CXCL6-forward | AGAGCTGCGTTGCACTTGTT | qRT-PCR |
| Human CXCL6-reverse | GCAGTTTACCAATCGTTTTGGGG | qRT-PCR |
| Human CXCL8-forward | TTTTGCCAAGGAGTGCTAAAGA | qRT-PCR |
| Human CXCL8-reverse | AACCCTCTGCACCCAGTTTTC | qRT-PCR |
| Human MMP3-forward | CGGTTCCGCCTGTCTCAAG | qRT-PCR |
| Human MMP3-reverse | CGCCAAAAGTGCCTGTCTT | qRT-PCR |
| Human SOX5-forward | CAGCCAGAGTTAGCACAATAGG | qRT-PCR |
| Human SOX5-reverse | CTGTTGTTCCCGTCGGAGTT | qRT-PCR |
| Human GAPDH-forward | GGAGCGAGATCCCTCCAAAAT | qRT-PCR |
| Human GAPDH-reverse | GGCTGTTGTCATACTTCTCATGG | qRT-PCR |
| Human SDC1-forward | AAAAGGGCGACTGTGGTTCA | CUT&Tag-qPCR |
| Human SDC1-reverse | AAGCATTAGGCAGTGGCTGT | CUT&Tag-qPCR |
| Human CXCL5-forward | CTCCCCACCAGTTCCCATTG | CUT&Tag-qPCR |
| Human CXCL5-reverse | CGGAGATTGGAGGAGCGAAG | CUT&Tag-qPCR |
| Human GAS1-forward | GACGGAATAAATGGGCACGC | CUT&Tag-qPCR |
| Human GAS1-reverse | GCAGAGCAGCCTCCCTTAAA | CUT&Tag-qPCR |
| Human CYP1B1-forward | CTCCGAGTAGTGGCCGAAAG | CUT&Tag-qPCR |
| Human CYP1B1-reverse | TAGTGGTGCTGAATGGCGAG | CUT&Tag-qPCR |

**Table S3 Characteristics of the study subjects receiving spine surgery**

|  | AS patients | non-AS patients |
| --- | --- | --- |
| Number | 6 | 6 |
| Age, year | 49.4±11.1 | 43.7±14.9 |
| Sex, male/female | 5/1 | 5/1 |
| Disease duration | 9.1±4.0 years | 2.6±0.9 days |
| CRP, mg/L | 21.9±10.8 | 4.9±2.1 |
| ESR, mm/h | 36.1±14.2 | 10.3±4.1 |
| HLA-B27 positive no. (%) | 6 (100%) | / |
| BASDAI | 3.07±0.69 | / |

Mean±SD. AS, ankylosing spondylitis; CRP, C-reactive protein; ESR, erythrocyte sedimentation rate; HLA-B27, human leukocyte antigen B27; BASDAI, the bath ankylosing spondylitis disease activity index. Non-AS patients were patients with spine fracture.
